# *TURAN* and *EVAN* Mediate Pollen Tube Reception in *Arabidopsis* Synergids through Protein Glycosylation

**DOI:** 10.1371/journal.pbio.1002139

**Published:** 2015-04-28

**Authors:** Heike Lindner, Sharon A. Kessler, Lena M. Müller, Hiroko Shimosato-Asano, Aurélien Boisson-Dernier, Ueli Grossniklaus

**Affiliations:** Institute of Plant Biology & Zurich-Basel Plant Science Center, University of Zurich, CH-8008 Zürich, Switzerland; University of California, Riverside, UNITED STATES

## Abstract

Pollen tube (PT) reception in flowering plants describes the crosstalk between the male and female gametophytes upon PT arrival at the synergid cells of the ovule. It leads to PT growth arrest, rupture, and sperm cell release, and is thus essential to ensure double fertilization. Here, we describe *TURAN* (*TUN*) and *EVAN* (*EVN*), two novel members of the PT reception pathway that is mediated by the FERONIA (FER) receptor-like kinase (RLK). Like *fer*, mutations in these two genes lead to PT overgrowth inside the female gametophyte (FG) without PT rupture. Mapping by next-generation sequencing, cytological analysis of reporter genes, and biochemical assays of glycoproteins in RNAi knockdown mutants revealed both genes to be involved in protein N-glycosylation in the endoplasmic reticulum (ER). *TUN* encodes a uridine diphosphate (UDP)-glycosyltransferase superfamily protein and *EVN* a dolichol kinase. In addition to their common role during PT reception in the synergids, both genes have distinct functions in the pollen: whereas *EVN* is essential for pollen development, *TUN* is required for PT growth and integrity by affecting the stability of the pollen-specific FER homologs ANXUR1 (ANX1) and ANX2. ANX1- and ANX2-YFP reporters are not expressed in *tun* pollen grains, but ANX1-YFP is degraded via the ER-associated degradation (ERAD) pathway, likely underlying the *anx1/2*-like premature PT rupture phenotype of *tun* mutants. Thus, as in animal sperm–egg interactions, protein glycosylation is essential for the interaction between the female and male gametophytes during PT reception to ensure fertilization and successful reproduction.

## Abstract 2

Protein glycosylation is essential for gametophyte interactions between the male pollen tube and the female ovule in plants, reminiscent of gamete interactions during fertilization in mammals.

## Author Summary

In flowering plants, gametes are produced by the haploid, multicellular male (pollen), and female (embryo sac) gametophytes, which develop within the reproductive organs of the flower. Successful fertilization depends on delivery of the sperm cells to the embryo sac, which is embedded in the ovule, by the pollen tube. Upon arrival of the pollen tube at the opening of the ovule, crosstalk between male and female gametophytes, known as pollen tube reception, ensues; the pollen tube slows or stops its growth, then resumes rapid growth, and finally bursts to release the sperm cells and effect double fertilization. Although several members of the pollen tube reception pathway, including the receptor-like kinase FERONIA, have been identified, the molecular mechanisms underlying this communication process remain unclear. Here, we show that protein N-glycosylation is required for normal pollen tube reception. A mutant screen identified two genes, *TURAN* and *EVAN*, which are involved in protein N-glycosylation in the endoplasmic reticulum. Both genes act in the FERONIA-mediated pollen tube reception pathway, which is impaired in these mutants. Thus, in plants, a “dual recognition system,” involving interactions between both protein and glycosyl residues on the surface of male and female gametophytes, appears to be required for successful pollen tube reception, conceptually similar to sperm–egg interactions in mammals, for which N-glycosylation of cell surface proteins also plays an important role.

## Introduction

In flowering plants, male and female gametes are constituents of the male (pollen) and female gametophytes (FG, embryo sac). A complex series of communication events between the male gametophyte and the female tissues of the flower is required for the pollen tube (PT) to deliver the two immotile sperm cells to the FG [1]. During double fertilization, one sperm cell each fuses with the egg cell and central cell to give rise to the embryo and endosperm, respectively. To reach the ovule-embedded FG, the PT grows through the stigma, the style, and the transmitting tract, enters the ovary, and grows along the funiculus towards the ovule. Gradients of various small, organic molecules [2,3], as well as larger peptides produced by the synergids [4], play an essential role in directing the PT to the FG. The synergids flank the egg cell at the micropylar end of the FG and secrete LUREs, small defensin-like proteins (DEFLs), which form a subgroup of cysteine-rich polypeptides (CRPs) [4–6]. Transduction of these female signals in the PT involves two receptor-like cytoplasmic kinases, LOST IN POLLEN TUBE GUIDANCE1 (LIP1) and LIP2 [7]. After arrival of the PT at the micropyle, it grows beyond the filiform apparatus (FA), a membrane-rich structure at the micropylar pole of the synergids, enters the receptive synergid, and ruptures to release the sperm cells [8]. Therefore, interactions between the PT and the synergids might consist of two spatially and temporally distinct stages. The first being PT reception at the FA, where PT growth is temporally slowed down or arrested, and the second involving rapid growth towards the PT entry site, PT rupture, and release of the two sperm cells with the concomitant death of the receptive synergid [9].

FERONIA (FER), a receptor-like kinase (RLK) of the *Catharanthus roseus* receptor-like kinase 1-like (*Cr*RLK1L) subfamily [10,11], is localized to the FA [12]. In mutants lacking FER activity, synergid development is normal, but *fer* (or *sirène* [13], which is allelic) mutant FGs remain unfertilized, even by wild-type PTs. The *fer* mutant thus revealed that an active signaling process is required for PT reception [10,12,13]. In these unfertilized ovules, the PT enters the receptive synergid but neither stops its growth nor ruptures to release the sperm cells. Instead, PT growth continues inside the FG leading to a PT overgrowth phenotype [10,12,13]. The two closest homologs of *FER* are the pollen-specific genes *ANXUR1* (*ANX1*) and *ANX2* [14,15]. *anx1* and *anx2* single mutants have no phenotype, but *anx1/2* double mutant PTs burst in vitro and in vivo shortly after germination [14,15]. Signaling via ANX proteins activates NADPH oxidases that produce reactive oxygen species (ROS) [16]. They fine-tune the Ca^2+^-gradient at the PT tip, resulting in the sustained secretion of membrane and cell wall material required for steady PT elongation [16]. However, upon PT arrival at the FG, *FER*-dependent accumulation of ROS around the FA leads to PT rupture [17]. Thus, the two ANX RLKs seem to ensure PT integrity until its arrival at the FA, and activation of the *FER*-dependent PT reception pathway leads to ROS accumulation and PT rupture [14,15,17].

Recently, binding of the 5kDa small peptide RAPID ALKANIZATION FACTOR1 (RALF1) to FER was shown in roots, where it leads to phosphorylation of plasma-membrane H^+^-ATPase 2 that regulates cell elongation [18]. However, it remains unclear whether pollen-expressed RALF-like proteins bind to FER in the synergids and activate the PT reception signaling cascade. In addition to FER, the following factors were shown to play a role in PT reception: LORELEI (LRE), a synergid-expressed glycosylphosphatidylinositol (GPI)-anchored protein [19], and NORTIA (NTA), a mildew-resistance locus O (MLO) family protein that accumulates at the FA in a *FER*-dependent manner upon PT arrival [20]. *lre-1/LRE* and *nta-1/nta-1* mutants show the *fer*-like PT overgrowth phenotype in 28% and 22% of the ovules, respectively [19,20]. Furthermore, the *abstinence by mutual consent* (*amc*) mutant, which affects a peroxin involved in protein import into peroxisomes, shows the *fer*-like phenotype only if mutant PTs contact mutant FGs [21]. This particular phenotype suggests a disrupted communication between both gametophytes due to missing signaling molecules from peroxisomes, possibly ROS. Recently, the first male gametophytic factors affecting PT reception were identified [22,23]. PTs of a triple mutant disrupting three MYB transcription factors (*myb97-1*, *myb101-4*, and *myb120-3*) fail to rupture and release the sperm in 60–70% of targeted ovules [22,23]. However, the target genes of these transcription factors, which may be involved in signaling, remain to be identified.

Here, we describe two novel mutants impaired in PT reception, *turan (tun)* and *evan (evn)*. Interestingly, *tun/TUN* and *evn/EVN* plants show the same female gametophytic *fer*-like PT overgrowth phenotype but have distinct pollen defects. Whereas *evn* mutant pollen grains degenerate before maturation, *tun* mutant grains develop normally, but PTs burst immediately after in vitro germination, reminiscent of the *anx1/2* phenotype. *TUN* and *EVN* are both involved in protein N-glycosylation in the endoplasmic reticulum (ER) and encode a putative uridine diphosphate (UDP)-glycosyltransferase superfamily protein and a dolichol kinase, respectively. The mutants do not affect the abundance and subcellular localization of FER, NTA, and LRE fusion proteins, suggesting that aberrant glycosylation may affect the function of at least one of these membrane proteins. In *tun* mutant pollen grains, ANX1 fused to the yellow fluorescent protein (ANX1-YFP) is degraded by the ER-associated degradation (ERAD) pathway. This leads to premature PT rupture, indicating that the ANX1/2 RLKs are targets of TUN-dependent N-glycosylation.

## Results

### *tun* and *evn* Mutants Show a *fer*-like Pollen Tube Overgrowth Phenotype

**Fig 1:**
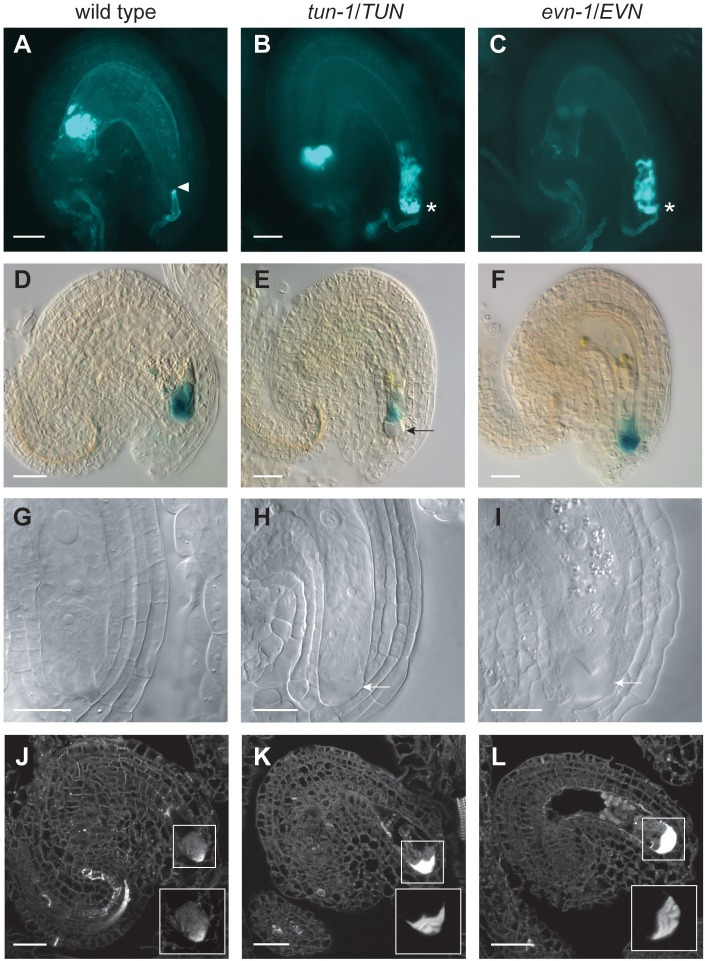
*tun* and *evn* ovules display pollen tube overgrowth and increased callose accumulation at the filiform apparatus. (A–C) Aniline Blue staining of callose in PT cell walls 2 d after pollination (DAP). (A) PT reception in a wild-type FG. Arrowhead indicates site of PT growth arrest. (B–C) PT overgrowth in *tun-1* (B) and *evn-1* mutant FGs (C). Asterisks indicate PT overgrowth. (D–F) β-glucuronidase (GUS) staining of synergid marker ET2634 2 d after emasculation (DAE) in wild-type (D), *tun-1* (E), and *evn-1* mutant FGs (F). Arrow indicates abnormal structure at the FA. (G–I) Chloral hydrate clearings of ovules 2 DAE in wild-type (G), *tun-1* (H), and *evn-1* mutants (I). Arrows indicate abnormal structure at the FA. (J–L) Aniline Blue staining of callose in 6 μm sections of wild-type (J), *tun-1* (K), and *evn-1* ovules 2 DAE (L). Boxes represent close-ups of indicated regions, whereby mutant close-ups in (K) and (L) were captured with reduced exposure time compared to the wild type (J). Scale bars in A–F and J–L = 20 μm; scale bars in G–I = 10 μm.

To gain more insight into the molecular mechanisms involved in PT reception in *Arabidopsis*, we conducted a forward genetic screen that yielded several mutants showing a *fer*-like PT overgrowth phenotype. The three mutants with the highest penetrance were chosen for further characterization, but two turned out to be allelic to each other (see below). Like *fer*, both mutants were named after Etruscan goddesses of fertility and fate, namely *turan (tun)* and *evan (evn)* [24]. In *tun-1/TUN*, *evn-1/EVN*, and *evn-2/EVN* heterozygous mutants 12% (*n* = 1,318), 20% (*n* = 1,233), and 22% (*n* = 320) of the ovules remained unfertilized, respectively, compared to only 1.5% (*n* = 1,389) in wild-type plants (Table 1). In unfertilized ovules, the PT continued to grow inside the FG, failed to arrest its growth, and did not rupture to release the sperm cells (Fig 1A–1C and S1C Fig).

**Table 1:** Overview of phenotypes in *tun* and *evn* mutant plants.

	Mutagen	Pollen tube overgrowth		Pollen phenotype		Transmission efficiency	
		%	n	%	n	female	male
wild type (Col-0)	NA	1.5%	1389	5% ± 5%^b^	128	NA	NA
*tun-1/TUN*	EMS	12%	1318	63% ± 1.5%^b^	441	74.5%	0%
*tun-2/TUN*	T-DNA	15%	513	44% ± 6.5%^b^	404	41.2%	0%
*evn-1/EVN*	EMS	20%	1233	54% ± 5%^c^	800	28%	0%
*evn-2/EVN*	EMS	22%	320	50% ± 0%^c^	200	27.8%	0%
*evn-3/EVN*	T-DNA	28%	337	50% ± 0%^c^	495	30%	0%
*tun-2/TUN; pTUN::TUN-GFP* ^a^	T-DNA	1%	280	1.7% ± 0.5%^b^	509	NA	NA
*TUN/TUN; pTUN::TUN-GFP* ^a^	NA	0%	320	3.6% ± 1.6%^b^	620	NA	NA

^a^ *pTUN::TUN-GFP* is homozygous

^b^ *anx1/2*-like pollen burst phenotype was assessed

^c^ *evn*-dependent pollen degeneration was assessed

NA, not applicable

To ensure that the PT overgrowth phenotype was not caused by cell specification defects in these mutants, a β-glucuronidase (GUS) synergid fate marker (ET2634) was analyzed. In both *tun* and *evn* mutant ovules, GUS expression was restricted to the synergids (Fig 1D–1F), indicating that their identity was not affected. However, some ovules showed an abnormal structure at the micropylar pole of the synergids (Fig 1E). To further investigate this structure, ovule membrane staining, clearings, and sections were analyzed 2 d after emasculation (DAE). Although membrane staining revealed no change in overall synergid morphology in the mutants (S2 Fig and S1 Data), approximately 50% of the mature FGs in cleared ovules of *tun-1/TUN* and *evn-1/EVN* mutants showed the abnormal structure in the FA region (Fig 1G–1I). Using Aniline Blue staining on ovule sections, we found that approximately 50% of the mature FGs showed increased callose deposition at the micropylar pole in both mutants (Fig 1J–1L). However, this did not influence PT attraction and reception, since all ovules could attract PTs, and over 60% of mutant FGs were fertilized. To investigate whether callose deposition in *tun* and *evn* FGs is an indicator of a defense-related response [25], expression of several plant defense pathway genes was tested in mutant pistils 2 DAE, but no up-regulation was observed (S3 Fig).

In summary, we identified two novel members of the PT reception pathway in the synergids, which are required for successful reproduction. In both mutants, synergid differentiation is normal, but callose accumulates at the micropylar pole of mutant FGs that, however, does not mediate the failure in PT reception.

### *tun* and *evn* Mutants Show Additional but Distinct Male Gametophytic Defects

**Fig 2:**
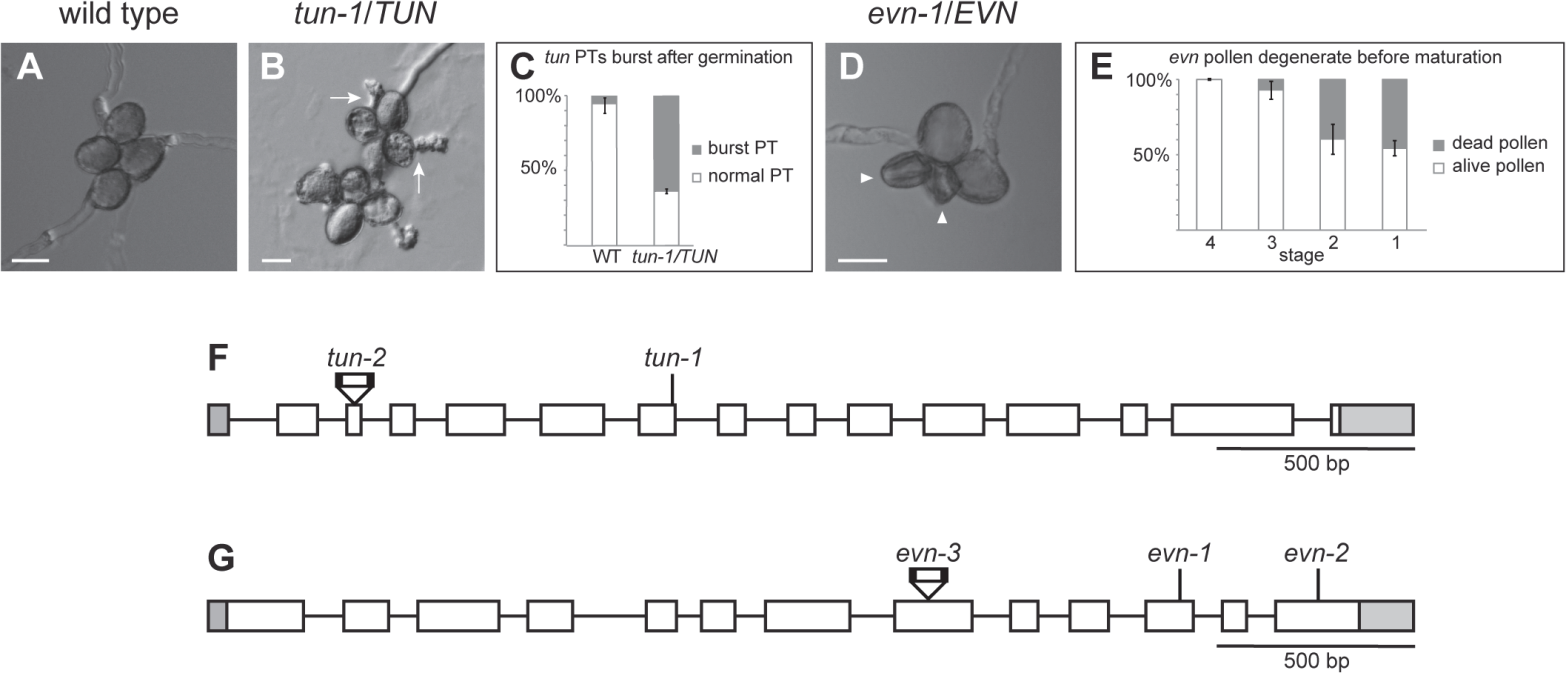
Distinct pollen defects in *tun* and *evn* mutants. (A) Pollen in vitro germination assay of *qrt/qrt* pollen grains. (B) Pollen in vitro germination assay of *tun-1/TUN;qrt/qrt* pollen. Arrows indicate PT bursting. (C) Graph of PT bursting counts in *qrt/qrt* and *tun-1/TUN;qrt/qrt* pollen. (D) Pollen in vitro germination assay of *evn-1/EVN;qrt/qrt* mutant pollen. Arrowheads indicate degenerated pollen grains. (E) Graph of degenerating pollen grain counts at different stages of *evn-1/EVN* mutants after DAPI staining. Stage four refers to the bicellular and early tricellular, stage three to the tricellular, stage two to the late tricellular and early mature, and stage one to the mature pollen stage. Scale bars: 20 μm. (F–G) Gene model of *TUN* (F) and *EVN* (G) with mutant alleles. Ethane methyl sulfonate (EMS) single nucleotide polymorphisms (SNPs) are indicated by lines, T-DNA insertions by triangles.

Self-pollination of both *tun-1/TUN* and *evn-1/EVN* mutant plants yielded only heterozygous and wild-type offspring. Attempts to propagate the mutants by crossing wild-type plants with mutant pollen produced no mutant progeny either (*n* = 96 plants per mutant; Table 1). These results suggest that not only the female but also the male gametophyte is affected in the *tun* and *evn* mutants. To further investigate this hypothesis, *tun-1/TUN* and *evn-1/EVN* plants were crossed to *quartet* (*qrt/qrt)* mutants [26,27], where microspores fail to separate after meiosis, forming tetrads of pollen grains. Heterozygous mutants in a *qrt/qrt* background facilitate the analysis of pollen defects because, within one tetrad, two microspores carry the wild-type and two the mutant allele. In vitro pollen germination experiments revealed that in *tun-1/TUN* mutants, pollen development was normal, but 63% ± 1.5% (*n* = 441) of mature PTs burst immediately after germination, compared to 5% ± 5% (*n* = 128) in the wild type (Fig 2A–2C and Table 1). This pollen phenotype of *tun* is reminiscent of double mutants disrupting *ANX1/2*, the pollen-specific homologs of *FER* [14,15]. In contrast to the wild type, where nearly all pollen were intact at maturity (*n* = 600), 54% ± 5% (*n* = 800) of the pollen grains degenerated before maturation in *evn-1/EVN* mutants, indicating general defects in pollen development (Fig 2D and 2E and Table 1). 4',6-diamidino-2-phenylindole (DAPI) staining of different developmental stages revealed that *evn* mutant pollen degenerates during the early tricellular stage (*n* = 2,816; Fig 2E and S4G Fig). Mutant pollen grains completed the second mitosis to form tricellular pollen, but comparison with the wild-type pollen within the same tetrad revealed a developmental delay in some cases (S4G Fig). Thus, pollen maturation seems disrupted in pollen grains lacking *EVN* activity.

Reciprocal crosses between *tun-1/TUN* and *evn-1/EVN* mutants with Col-0 wild-type plants showed that the *fer*-like PT overgrowth phenotype was caused by a female gametophytic defect. Whereas mutant ovules fertilized by wild-type pollen showed the *fer*-like PT overgrowth phenotype, wild-type pistils pollinated with pollen from heterozygous mutants showed no phenotype (S5 Fig) as expected, since no functional mutant PTs are formed. Both *evn* and *tun* pollen defects were fully penetrant (i.e., affecting male gametophyte viability and PT growth, respectively) with a male transmission efficiency of 0% (*n* = 96 plants per mutant; Table 1). In contrast, female transmission efficiency was reduced to 74.5% (*n* = 96) and 28% (*n* = 96) in *tun-1/TUN* and *evn-1/EVN* mutants, respectively (Table 1).

Taken together, these results show that *tun* and *evn* mutants display distinct male gametophytic defects, affecting male gametophyte growth and viability, respectively. However, abnormal PT reception is caused by a female gametophytic defect, leading to the decreased female transmission of the mutant alleles.

### *TUN* Encodes a Putative UDP-Glycosyltransferase and *EVN* a Dolichol Kinase

In order to identify the causative mutations, we developed SNP-ratio mapping (SRM) [28]. SRM enables the mapping of heterozygous mutant individuals by next-generation sequencing. Briefly, the segregation ratios of EMS-induced SNPs are used to identify the causative SNP that segregates in a 1:1 ratio in a backcross mutant population versus a 1:3 ratio for unlinked SNPs segregating in the background.

Applying SRM to *tun-1/TUN* mutants revealed a stop codon in the sixth exon of *At1g16570* (Fig 2F) [28], which encodes a putative UDP-glycosyltransferase superfamily protein belonging to the glycosyltransferase (GT) family 33, whereof *TUN* is the only member in *Arabidopsis*. In *evn-1/EVN* mutants, SRM identified a stop codon in the eleventh exon of *At3g45040* (Fig 2F and S6 Fig and S1 Table), which encodes the only dolichol kinase in the *Arabidopsis* genome [29]. Thus, both *TUN* and *EVN* encode proteins that are likely playing roles in protein N-glycosylation. Whereas EVN as the only dolichol kinase may have a general role, TUN potentially acts in a more specific manner, since a total of 27 GT families have been identified in *Arabidopsis*. Finally, a second allele of *evn* (*evn-2*) was found in the same EMS screen and identified by a combination of SRM (S7 Fig) and classical map-based cloning (S1 Text). This allele has a premature stop codon in the last exon and displays similar PT overgrowth (22%; *n* = 320) and pollen phenotypes (50% ± 0%; *n* = 200) as *evn-1* (Fig 2F and Table 1 and S1C Fig, S1G Fig and S7 Fig and S2 Table).

To confirm that the correct genes had been identified, T-DNA insertion lines disrupting them were analyzed. As described previously, the T-DNA allele *tun-2* (SAIL_400_A01) has an insertion in the fourth exon of *At1g16570* and displays a PT overgrowth phenotype (15%; *n* = 513; Fig 2F and Table 1 and S1B Fig) [28] and PT bursting in vitro (44% ± 6.5%; *n* = 404; Table 1 and S1F Fig) similar to the EMS allele *tun-1*, confirming the correct identification of *TUN* by SRM. Likewise, *evn-3* (SAIL_529_E06) has an insertion in the eighth exon of *At3g45040* and shows the same female (28%; *n* = 337) and male (50% ± 0%; *n* = 495) [29] phenotypes as the EMS mutants *evn-1* and *evn-2* (Fig 2G and Table 1 and S1D Fig and S1H Fig).

In summary, *TUN* and *EVN* both encode proteins potentially involved in protein N-glycosylation, suggesting that proper N-glycosylation of proteins involved in PT reception is critical for the gametophytic dialogue during PT reception.

### TUN and EVN Are Involved in Protein N-Glycosylation, and Down-Regulation Causes Distinct Vegetative Phenotypes

N-linked protein glycosylation occurs as proteins transit through the ER [30]. To gain insight into the subcellular localization of the TUN and EVN proteins, and to confirm their identity by functional complementation, native promoter::protein-green fluorescent protein (GFP) fusions were transformed into mutant plants. The *pTUN::TUN-GFP* construct complemented both the female and the male phenotypes (Table 1). In ovules, the strongest GFP signal was observed in the FG including the synergids, which showed a ring shaped signal around their nuclei, and throughout the PT (S8A–S8C Fig). The *pEVN::EVN-GFP* construct neither showed GFP expression nor complemented the phenotypes, suggesting that the construct was not functional in planta, likely due to missing regulatory elements.

Colocalization studies of *p35S::TUN-GFP* and *p35S::EVN-GFP*, where the fusion proteins were expressed under the constitutive, viral 35*S* promoter, with different subcellular markers were performed in transiently transformed onion and tobacco epidermis cells. They showed ER localization of both proteins (S8D and S8E Fig), supporting potential roles of TUN and EVN in protein N-glycosylation.

**Fig 3:**
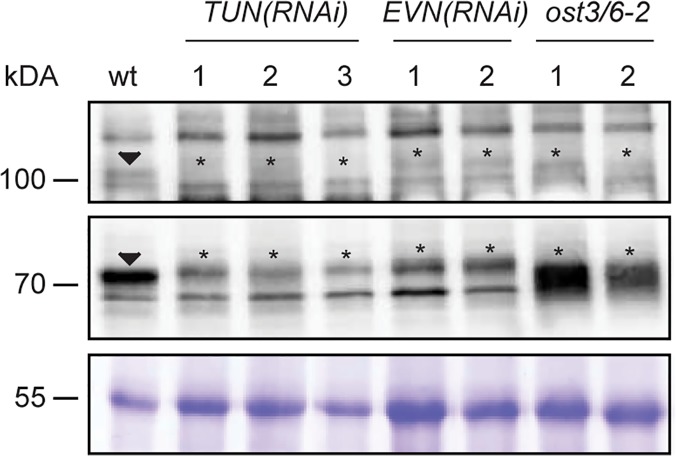
ConA reveals altered glycoprotein patterns in *TUN(RNAi)* and *EVN(RNAi)* seedlings. Lectin blot using ConA of a wild-type, three independent *TUN(RNAi)* lines, two independent *EVN(RNAi)* lines, and two *ost3/6-2* control plants. Arrowheads indicate wild-type bands with differential abundance and/or mobility in knockdown lines, marked by asterisks. 55kDa fraction represents a Commassie-Brilliant Blue stained loading control.

As no homozygous mutants could be recovered, we used RNA interference (RNAi) to down-regulate expression of *TUN* and *EVN* using the 35*S* promoter. Four independent *TUN(RNAi)* and *EVN(RNAi)* lines with greatly reduced expression, down to 12% and 13% of wild-type levels, respectively (S9 Fig and S1 Data), were chosen for further analysis. To investigate the function of *TUN* and *EVN* in a biochemical analysis of glycoproteins, potential changes in glycoprotein abundance in knockdown seedlings were assessed by a lectin blot using Concanavalin A (ConA), which mainly binds to terminal mannosyl and glucosyl residues of glycoproteins. Both, *TUN(RNAi)* and *EVN(RNAi)* seedlings showed altered glycoprotein abundance and mobility compared to both the wild type and *ost3/6-2*, a mutant disrupted in a subunit of an oligosaccharyltransferase that acts later in the glycosylation pathway (Fig 3) [31].

**Fig 4:**
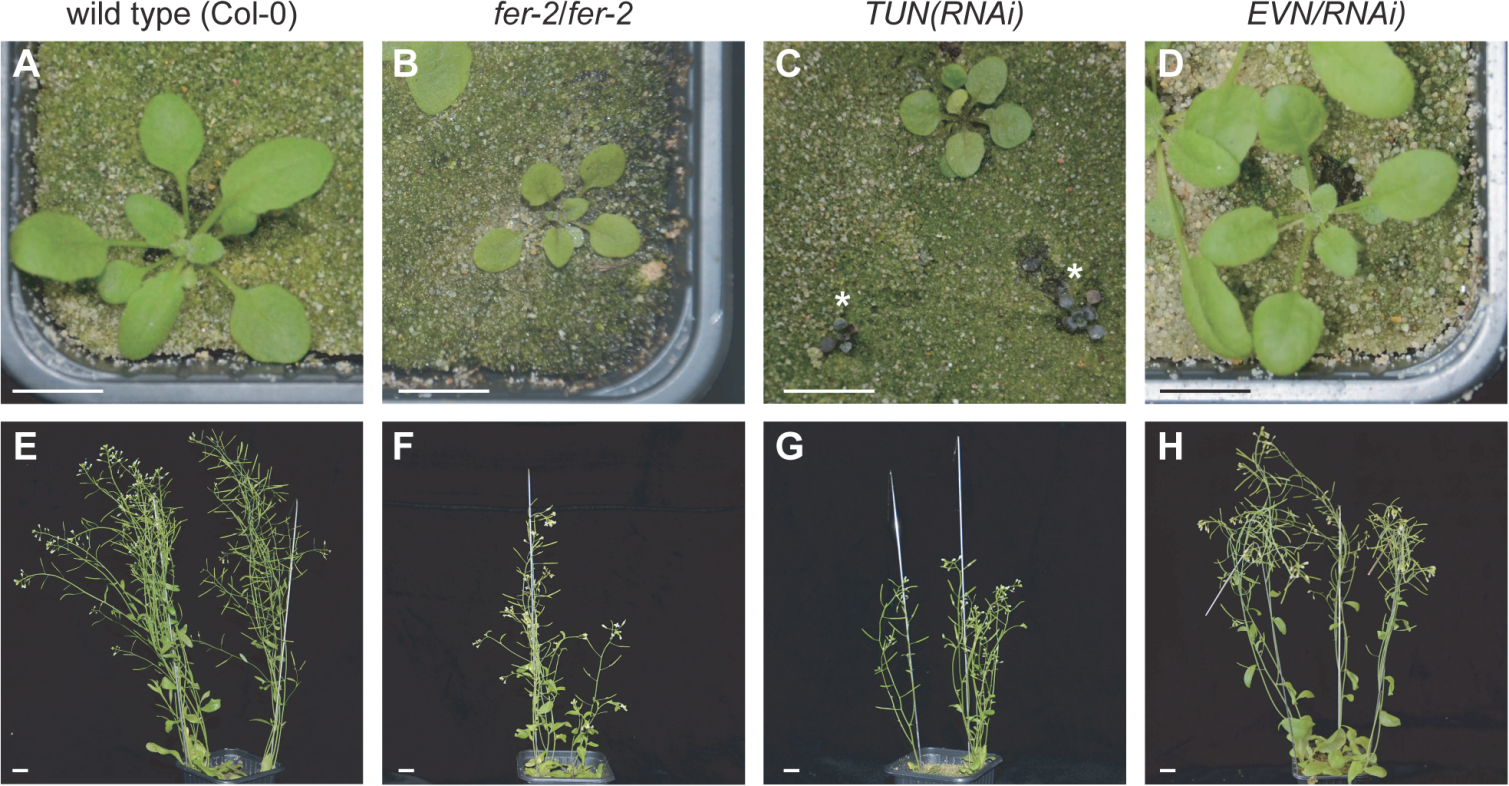
*TUN(RNAi)* lines show *fer*-like vegetative dwarf phenotype. (A–D) Plant size of 30-d-old seedlings of wild-type (A), *fer-2/fer-2* (B), *TUN(RNAi)* (C), and *EVN(RNAi)* lines (D). Asterisks indicate *TUN(RNAi)* seedlings, that accumulate athocyanins and degenerate without further growth. (E–H) Plant size of adult wild-type (E), *fer-2/fer-2* (F), *TUN(RNAi)* (G), and *EVN(RNAi)* (H) plants. (F) Left plant is at the same developmental stage as wild-type, *TUN(RNAi)*, and *EVN(RNAi)* individuals. Scale bars: 1 cm. All lines are in the Col-0 background.

Furthermore, *TUN(RNAi)* knockdown lines displayed a vegetative phenotype reminiscent of *fer/fer* mutants (Fig 4) [20,32]. The severity and frequency of the dwarf phenotype correlated with the expression level of *TUN* in the *TUN(RNAi)* lines (Fig 4C and 4G and S9 Fig). Seedlings on plates looked normal, but in soil, some plants remained small, accumulated anthocyanins, and eventually died without further growth (Fig 4C). It is possible that *TUN* expression in these seedlings was very low, but it could not be determined due to early lethality. These results indicate that *TUN* is essential for vegetative development. In contrast, *EVN(RNAi)* knockdown plants showed no obvious phenotype (Fig 4D and 4H and S9 Fig), suggesting that low levels of *EVN* are sufficient to support normal vegetative growth.

Taken together, both TUN and EVN play a role in protein N-glycosylation but only *TUN* seems required for normal vegetative growth and development.

### Down-Regulation of *TUN* Does Not Cause Deglycosylation of FER

Protein N-glycosylation plays important roles in many processes, including protein folding, protein stabilization, protein targeting [33], and receptor–ligand interactions [34]. In *tun* mutants, we observed *fer*-like PT overgrowth, a *fer*-like vegetative phenotype, and *anx1/2*-like PT bursting. The FER, ANX1, and ANX2 proteins have multiple predicted glycosylation sites [11], indicating that TUN may be involved in the specific glycosylation of this RLK subfamily. To investigate whether FER requires *TUN* for glycosylation, a *TUN(RNAi)* construct was transformed into plants expressing a *pFER::FER-GFP* translational fusion protein. In the case of a complete absence of potential N-glycans attached to FER, we would expect a smaller size of the under-glycosylated FER protein in *TUN(RNAi)* seedlings compared to the wild type [31]. However, no obvious difference in size, indicating a loss of glycosylation, was detected between FER-GFP in *TUN(RNAi)* and wild-type seedlings by immunoblot analysis using an anti-GFP antibody (S10 Fig). In contrast, a clear shift in size was seen when FER-GFP was deglycosylated by Endoglycosidase H (EndoH, a high mannose N-glycan deglycosylase) treatment in vitro (S10 Fig).

These data suggest that FER-GFP is not completely deglycosylated in *TUN(RNAi)* seedlings. However, we cannot exclude the possibility that residual levels of TUN activity in the RNAi lines were able to partially glycosylate FER in seedlings, or that FER-GFP is misglycosylated rather than deglycosylated.

### FER, NTA, and LRE Are Not Mislocalized in *tun* and *evn* Mutant Embryo Sacs

**Fig 5:**
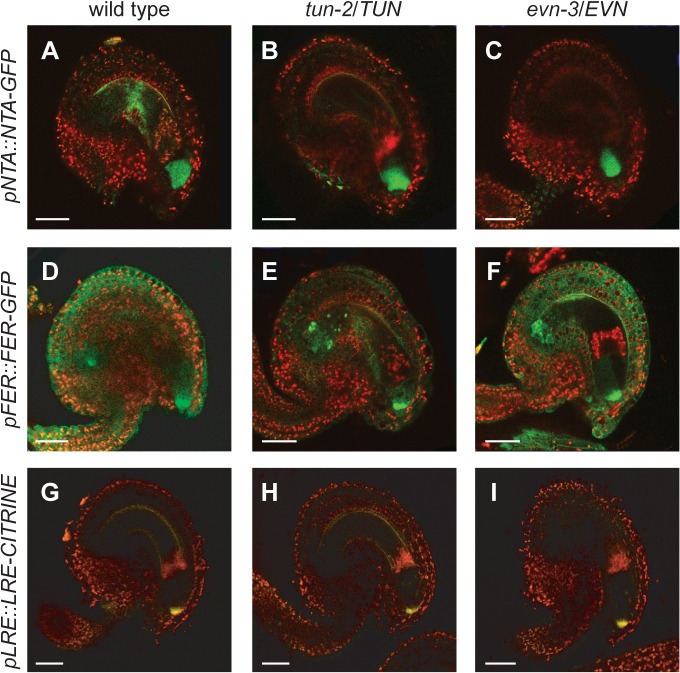
NTA, FER and LRE show proper localization in *tun* and *evn* mutant embryo sacs. (A–I) Confocal microscope analysis of fluorescently labeled proteins. (A–C) Vesicle-associated NTA-GFP localization in the cytoplasm of a wild-type (A), *tun-2* (B), and *evn-3* FG (C). (D–F) FER-GFP at the FA and in membranes of sporophytic tissue of a wild-type (D), *tun-2* (E), and *evn-3* FG (F). (G–I) Extracellular localization of LRE-Citrine in a wild-type (G), *tun-2* (H), and *evn-3* FG (I). Scale bars: 20 μm.

Since *tun* and *evn* show *fer*-like PT overgrowth, we hypothesized that the stability and/or localization of known female players involved in PT reception could be compromised in *tun* and *evn* mutant FGs. Therefore, FER-GFP, NTA-GFP, and LRE-Citrine reporter constructs were introduced into the *tun* and *evn* mutant backgrounds and analyzed for changes in expression and localization. First, *pFER::FER-GFP* and *pNTA::NTA-GFP* translational fusions were analyzed in *tun-2/TUN* and *evn-3/EVN* siliques 2 DAE. Whereas NTA-GFP was shown to localize in vesicle-like structures throughout the cytoplasm before fertilization [20], FER-GFP is localized to the FA in the synergids [12]. Neither NTA-GFP nor FER-GFP localization was changed in *tun* and *evn* mutants (Fig 5A–5F and S11 Fig and S1 Data), indicating that PT overgrowth in *tun* and *evn* is not caused by mislocalization of FER and NTA.

Although no reporter gene was available for *LRE*, encoding a predicted GPI-anchored protein, in situ hybridization showed *LRE* expression predominantly in synergids before fertilization [19]. A LRE-GFP fusion protein did not produce a detectable fluorescent signal in transient transformations [19], possibly due to the exposure of GFP to the acidic pH in the apoplast. However, LRE is a good candidate for a target of EVN because the yeast mutant corresponding to *evn*, *secretory59 (sec59)*, is depleted in GPI-anchored proteins [35]. Therefore, we produced a LRE reporter using the pH-stable fluorescent protein Citrine [36], and cloned it between the predicted signal peptide and GPI-anchor of *LRE* [19]. The LRE-Citrine fusion protein localized to the surface of the synergids, appearing different from the FA localization of FER-GFP (Fig 5D and 5G). Thus, LRE-Citrine likely faces the extracellular space towards the micropyle, where PT reception is initiated upon PT arrival. However, LRE-Citrine production and localization were unaffected in *evn* and *tun* mutants (Fig 5G–5I and S11 Fig and S1 Data).

In summary, PT reception defects in *tun* and *evn* FGs are not caused by misexpression or mislocalization of FER, NTA, and LRE. However, it is possible that protein function is impaired due to misglycosylated residues—particularly in FER.

### ANX1-YFP and ANX2-YFP Are Not Detectable in *tun* Mutant Pollen Grains

**Fig 6:**
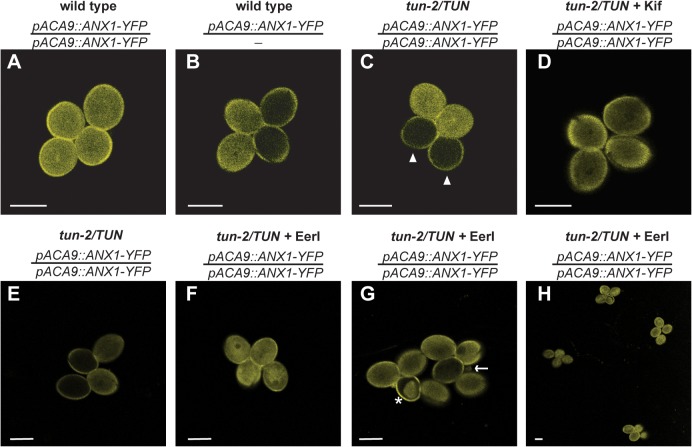
ANX1-YFP fluorescence is not detectable in *tun* mutant pollen grains. (A–D) Confocal microscope analysis of *ANX1-YFP* expression under a pollen-specific promoter. (A) *ANX1-YFP* expression in *TUN/TUN;qrt/qrt;ANX1-YFP/ANX1-YFP* (wild-type segregants homozygous for the reporter gene). (B) *ANX1-YFP* expression in *TUN/TUN;qrt/qrt;ANX1-YFP/-* (wild-type segregants hemizygous for the reporter gene). (C) *ANX1-YFP* expression in *tun-2/TUN;qrt/qrt;ANX1-YFP/ANX1-YFP* mutant tetrads. Arrowheads indicate missing fluorescence in *tun* pollen grains. (D) *ANX1-YFP* expression in *tun-2/TUN;qrt/qrt;ANX1-YFP/ANX1-YFP* mutant tetrads after Kifunensine (Kif) treatment. (E) *ANX1-YFP* expression in *tun-2/TUN;qrt/qrt;ANX1-YFP/ANX1-YFP* mutant tetrads after mock treatment for fluorescence intensity decrease comparison. (F–H) *ANX1-YFP* expression in *tun-2/TUN;qrt/qrt;ANX1-YFP/ANX1-YFP* mutant tetrads after Eeyarestatin I (EerI) treatment. (F) 10 μm EerI recovers ANX1-YFP fluorescence in *tun* pollen grains. (G) Higher concentrations of EerI can lead to cytosolic inclusions (asterisk) or pollen grain burst (arrow). (H) ANX1-YFP fluorescence recovery in several *tun-2/TUN;qrt/qrt;ANX1-YFP/ANX1-YFP* mutant tetrads after EerI treatment. Residual fluorescence signal from the pollen coat is autofluorescence. Scale bars: 20 μm.

To investigate whether the PT bursting phenotype of *tun* is caused by alterations in ANX1/2 protein abundance and/or localization, we transformed *tun-2/TUN;qrt/qrt* mutants with *pACA9::ANX1-YFP* and *pACA9::ANX2-YFP*, respectively, and crossed *tun-2/TUN;qrt/qrt* to plants expressing *pACA9::ANX1-YFP* [16]. We analyzed four independent lines homozygous for the *pACA9::ANX1-YFP* transgene (T2 or F2 generation) and two independent lines hemizygous for the *pACA9::ANX2-YFP* transgene (T1 generation) and heterozygous for *tun-2*, respectively. Although the *tun-2/TUN* mutants were homozygous for *pACA9::ANX1-YFP*, we always found ANX1-YFP expression in only two pollen grains per tetrad (Fig 6A–6C). However, in wild-type segregants homozygous *pACA9::ANX1-YFP* fluorescence could be observed (Fig 6A). Furthermore, *tun-2/TUN* mutants hemizygous for *pACA9::ANX2-YFP* displayed only 30.2% ± 0.7% (*n* = 156 tetrads) fluorescent pollen grains, compared to 50% ± 0% (*n* = 150 tetrads) in wild-type tetrads. Since only fluorescent tetrads were counted under the epifluorescence microscope, a reduction of 50% fluorescence of a hemizygous reporter would result in 33,3% fluorescent pollen grains.

Thus, ANX1-YFP and ANX2-YFP are not detectable in *tun* mutant pollen grains, possibly because they are subjected to ERAD of misfolded proteins [37]. Indeed, treatment with Kifunensine (Kif), an inhibitor of the ERAD pathway [38] resulted in the recovery of ANX1-YFP fluorescence in *tun* pollen grains (Fig 6D). Because the outcomes of Kif treatments were variable, likely dependent on how well the drug was taken up in different experiments, we also used the ERAD inhibitor Eeyarestatin I (EerI) [39], which resulted in a more consistent recovery of ANX1-YFP fluorescence (Fig 6F–6H and S12 Fig and S1 Data). These results indicate that the premature bursting of *tun* PTs is caused by the absence of ANX1 and ANX2.

Therefore, *TUN* seems to be involved in ANX1-YFP and ANX2-YFP glycosylation, and the aberrant glycosylation of these fusion proteins leads to their degradation by the ERAD pathway. However, although less likely, we cannot exclude that ANX1/2-YFP proteins get degraded because another protein required for their stability is misglycosylated and subject to the ERAD pathway.

## Discussion

### *TUN* and *EVN* Encode Proteins of the Early N-Glycosylation Pathway

In this study, we characterized two mutants, *tun* and *evn*, isolated in a screen for defects in PT reception. They have similar PT overgrowth phenotypes in the FG, but play distinct roles during pollen development, PT integrity, and vegetative growth. Both genes encode proteins that are involved in N-linked protein glycosylation, indicating that this cotranslational protein modification is essential to various developmental processes.

N-linked glycosylation affects protein folding, stability, transport, and activity [40]. It is a multistep process starting with the assembly of an oligosaccharide, containing N-acetylglucosamine (GlcNAc), mannose (Man), and glucose (Glc), on a phosphorylated membrane-bound polyisoprenoid lipid carrier, dolichol [30]. The assembly starts on the cytosolic side and finishes on the lumenal side of the ER, yielding the end product Glc_3_-Man_9_-GlcNAc_2_ [30]. The second step is the cotranslational transfer of the oligosaccharide to an asparagine in the Asn-X-Ser/Thr sequon, where X can be any amino acid except proline [41]. The last step is ER-associated quality control, which ensures proper folding of N-glycosylated proteins and their subsequent exit of the ER [37].

*EVN* encodes a dolichol kinase, which plays an early role in the N-glycosylation pathway [29]. The SEC59 dolichol kinase in yeast catalyzes the cytidine-triphosphate-dependent phosphorylation of ER-membrane-bound dolichol [35]. As *EVN* can complement the yeast *sec59* mutant, it seems to perform the same biochemical function [29]. Phosphorylated dolichol (Dol-P) serves as carrier for the assembly of the oligosaccharide on the cytosolic side of the ER and, additionally, as a carrier for the monosaccharides Man and Glc in the ER lumen for GPI-anchor synthesis. In *Arabidopsis*, *EVN* is a single copy gene with characterized orthologs in yeast [42], mammals [43,44], and humans [45], where dolichol kinase deficiency results in hypoglycosylation and lethality [46,47]. We never retrieved any homozygous mutants for *evn*, due to its complete male gametophytic lethality. As *EVN(RNAi)* knockdown plants do not show any obvious vegetative phenotype, a low, residual gene activity might be sufficient for normal plant growth.

*TUN* encodes a putative UDP-glycosyltransferase superfamily protein belonging to GT family 33, whereof TUN is the only member in *Arabidopsis*. The yeast ortholog *ALG1* encodes a beta-1,4-mannosyltransferase, which transfers the first Man from the GDP-D-Man substrate to the Dol-PP-GlcNAC_2_ acceptor on the cytosolic side of the ER [48,49]. ALG1 resides in the ER membrane, forming homodimers and heterodimers with ALG2 and ALG11, respectively [50]. Under nonpermissive conditions, temperature-sensitive *alg1-1* mutants produce no Man-containing oligosaccharides [48], leading to lethality [51]. As for *evn*, no homozygous *tun* mutants were recovered because of complete male gametophytic lethality. However, the strong dwarf phenotype and dying seedlings in *TUN(RNAi)* lines indicate that *TUN* is also required for vegetative growth and development. In plants, the importance of protein N-glycosylation during embryogenesis, vegetative development, and plant defense has been demonstrated by various phenotypes of N-glycosylation mutants [52].

The subcellular localization of TUN and EVN fusion proteins in the ER and the altered glycoprotein patterns observed in RNAi lines for both genes indicate that the plant proteins play similar roles in protein glycosylation as their yeast orthologs.

### Localization of Known Members of the Pollen Tube Reception Pathway Is Not Affected in *evn* and *tun* Synergids

We showed that mutations in *EVN* and *TUN* in the FG lead to PT reception defects. Due to their protein localization to the ER, it is likely that EVN and TUN do not directly mediate male–female gametophyte interactions during PT reception, but that an impaired glycosylation pathway affects the function of female players involved in this process. However, the localization of translational reporter gene fusions with FER, NTA, and LRE, three known components of the PT reception pathway, was unaffected in FGs of *evn-3/EVN* and *tun-2/TUN* plants. This was not surprising for NTA-GFP, which has no predicted glycosylation sites. However, LRE-Citrine fluorescence was expected to be reduced or even absent in *evn* mutant ovules, since the yeast *sec59* mutant is depleted in GPI-anchored proteins [35]. However, EVN and SEC59 display only 22% amino acid identity (ClustalW). Although the plant protein is able to complement the yeast *sec59* mutant [29], the two proteins may not play identical roles in planta and/or other, undescribed kinases could take over *EVN* function in mutant plants.

However, normal protein abundance and localization does not ensure proper function. The fact that *tun* mutants show *fer*-like PT overgrowth, a *fer*-like vegetative phenotype, and *anx1/2*-like PT bursting, suggests that the FER and ANX1/2 RLKs are affected in this mutant. FER has nine putative N-glycosylation sites, eight of which reside in the extracellular malectin-like domain [11]. In *Xenopus laevis*, malectin is an ER-localized lectin, which selectively binds carbohydrates and is involved in the ER-quality control of glycoproteins [53,54]. The extracellular, glycosylated malectin-like domain of FER suggests that its ligand could contain sugar residues and/or that proper glycosylation of this domain is essential for ligand binding. However, potential deficiencies in protein glycosylation in *tun* and *evn* mutant FGs did not affect polar localization of FER-GFP to the FA, and down-regulation of *TUN* did not cause a complete loss of N-linked glycans in FER-GFP in seedlings. But it is possible that only some N-glycans are absent or that N-glycan composition is altered. Therefore, while FER-GFP localization is unaffected, functions such as ligand binding and/or recognition and subsequent signal transduction could be affected. This has been shown to be the case for the plant immunity leucine-rich repeat (LRR) RLK *EF*-Tu receptor (EFR), which shows impaired ligand binding as a consequence of underglycosylation [34]. Just recently, binding of the 5kDa small peptide RALF1 to FER was shown in roots [18]. However, RALF1 is neither expressed in PTs nor in synergid cells. But there are 34 RALF-like peptides with various expression patterns throughout the plant [18]. In the case of RALF1, N-glycosylation does not seem to play a role in FER binding, since the processed peptide lacks any predicted N-glycosylation site. Out of seven highly pollen-expressed RALF-like peptides, RALF-like4 and RALF-like26 are the only two with a predicted N-glycosylation site. It is conceivable that, unlike in animals [53,54], the malectin-like ligand-binding domain of FER has no conserved carbohydrate binding capacity in plants. Instead, the glycosylation state of the ligand-binding domain of the receptor could be more important than glycosylation of the ligand.

PT reception at the FA is essential for double fertilization and thus successful reproduction. It is conceivable that a “dual recognition system” exists, where the protein backbone of the receptor is required for interactions with the ligand, which are further enhanced by N-linked glycans, thereby increasing the chances for successful PT reception. Such a dual recognition system has been described for gamete interactions in mammals [55], where the glycoprotein mZP3 in the egg cell’s extracellular matrix, the zona pellucida, is responsible for sperm binding [56]. While sperm binding is improved by glycosylated mZP3 proteins, unglycosylated mZP3 can also bind sperm. Accordingly, a sperm protein or protein complex interacts with the glycans and/or the protein backbone of mZP3 depending on its glycosylation state. Thus, this dual adhesion system ensures better sperm–egg binding but allows gamete interaction even if glycan composition is disturbed, increasing the chance for successful fertilization [57]. In plants, the existence of such a dual recognition system could explain the reduced penetrance of the PT overgrowth phenotype in *tun* and *evn* since the female protein component, even if not glycosylated properly, could still partially recognize the male ligand. Despite the similarities of the *fer* and *tun* phenotypes, further biochemical investigations are required to investigate whether indeed the FER RLK and/or other components of the PT reception pathway are affected in the *tun* mutant.

### ANX1-YFP Gets Degraded by the ERAD Pathway in *tun* Pollen Grains

Apart from the phenotypes it shares with *fer*, *tun* also shows *anx1/2*-like PT bursting. ANX1 and ANX2 have seven and four potential N-glycosylation sites, respectively [11], with all four predicted glycosylation sites of ANX2 being located in the extracellular domain and conserved in ANX1. ANX1-YFP and ANX2-YFP fluorescence is not detectable in *tun-2* mutant pollen grains, but ANX1-YFP fluorescence is recovered after treatment with two ERAD inhibitors. This finding suggests that ANX1-YFP is misglycosylated in *tun* pollen grains and is therefore degraded by the ERAD pathway, which is induced when N-glycan-dependent protein folding fails [37]. The misfolded protein is recognized by ubiquitin ligases, ubiquitinatinylated, retrotranslocated to the cytosol, and degraded by the proteasome [37]. For instance, the LRR-RLK BRASSINOSTEROID-INSENSITIVE1, which contains multiple N-glycosylation sites [58], is such an N-glycan-dependent ERAD target [59,60].

Since FER, NTA, and LRE fusion proteins did not show any alteration in protein abundance or localization in *tun* mutant ovules, the effect on ANX1-YFP and ANX2-YFP seems to be a pollen-specific effect rather than a general loss of glycosylated proteins in the plant. Specificity of ERAD has been described for the LRR-RLKs EFR and FLAGELLIN-SENSITIVE2 (FLS2), involved in plant innate immunity [61]. Both LRR-RLKs are N-glycosylated membrane proteins, but mutations in two members of the ER quality control pathway affected only EFR but not FLS2 [61]. Similarly, the closely related *Cr*RLK1Ls FER, ANX1, and ANX2 might all be misglycosylated in the *tun* mutant, but only ANX1/2 are degraded via the ERAD pathway, explaining the absence of ANX1/2-YFP but the normal abundance of FER-GFP. This would also explain the difference in the penetrance of male and female gametophytic phenotypes; although FER is likely misglycosylated, it is still localized to the FA, thereby allowing successful PT reception to a certain degree, consistent with the residual transmission of *tun* through the FG. In contrast, ANX1/2 are degraded by the ERAD pathway leading to a fully penetrant *anx1/2*-like phenotype and the complete absence of transmission through the pollen.

### Conclusions

The characterization of *TUN* and *EVN* demonstrated that protein N-glycosylation is important for various developmental processes. Impaired PT reception and *anx1/2*-like PT rupture in *tun* mutants appears to be linked to misglycosylated FER and ANX1/2, respectively, the most closely related members of the *Cr*RLK1L subfamily of RLKs. The defects in PT reception observed in the *tun* and *evn* glycosylation mutants provides a first indication that plants evolved similar mechanisms to ensure fertilization as mammals, where both N-glycan–protein and protein–protein interactions appear to act synergistically to guarantee gamete binding and, thus, enhance the chance for successful fertilization.

## Materials and Methods

### Plant Material and Growth Conditions

Plant growth conditions were as described [62]. T-DNA insertion lines were obtained from the Nottingham *Arabidopsis* Stock Center (NASC). The *qrt1-2/qrt1-2* mutant [26], was a gift from J. F. Harper (University of Nevada, Reno). The *pACA9::ANX1-YFP*, *pACA9::ANX2-YFP*, *pFER::FER-GFP*, and *pNTA::NTA-GFP* lines were described previously [12,16,20]. Crosses were done by emasculating wild-type Col-0, *tun/TUN* and *evn/EVN* flower buds, followed by pollination with respective pollen 2 DAE. Identification of the EMS alleles, cloning procedures, genotyping, and gene expression analysis are described in S1 Text.

### Aniline Blue Staining

To visualize PTs, siliques were selected around 2 DAP. Sepals and petals were removed, and siliques were fixed in 9:1 ethanol (EtOH):acetic acid overnight at 4°C. Aniline Blue staining was previously described [10], and sample analysis was done using a Leica DM6000B epifluorescence microscope. Callose staining of semithin ovule sections is described in S1 Text.

### In Vitro Pollen Germination Analysis

In vitro pollen germination was previously described [14]. Pollen grains and tubes were imaged using the differential interfering contrast on a Leica DM6000B microscope.

### FM4-64 Membrane Staining

Wild-type, *tun-2/TUN*, and *evn-3/EVN* pistils were dissected in ice cold 100 mM FM4-64 Dye (Life Technologies) solution on microscope slides, and ovules were covered with a cover slip. Microscope slides were covered with aluminum foil and left on ice for 3–4 h. Fluorescence of ovule membranes was analyzed using a Leica SP5 confocal microscope.

### Ovule Clearing

Flower buds were emasculated and collected 2 DAE, carved open longitudinally on the sides, and fixed in 9:1 EtOH:acetic acid over night at 4°C. Samples were washed in an EtOH series (85%, 70%, 50%, and 30% for 30 min each) and clearing solution (chloral hydrate:glycerol:water (8:1:2, w:v:v)) was added. Siliques were dissected and ovules were analyzed using a Leica DMR microscope.

### β-Glucuronidase (GUS) Staining

The synergid marker ET2634 [63] was crossed to *tun-1/TUN* and *evn-1/EVN* mutants. Homozygous F2 individuals were emasculated and siliques were stained for GUS expression 2 DAE as previously described [20]. Stained samples were dissected and analyzed using a Leica DMR microscope.

### Western Blot and Deglycosylation Analysis

Protein extraction from 10-d-old seedlings (approximately 400; wild-type, *tun-2/TUN*, and *evn-3/EVN* plants carrying *pFER::FER-GFP*, and *ost3/6-2* (SALK_067271)) was conducted by grinding them in a mixer mill and adding extraction buffer (50 mM Tris pH7.5, 10 mM NaCl, 0,5% Triton X-100, and a tablet of Complete Mini protease inhibitor cocktail (Roche)). Extracts were incubated on ice for 15 min and centrifuged for 3 min at 14,000 rpm (Eppendorf centrifuge 5424 with a FA-45-24-11). Protein extracts were boiled at 95°C with SDS loading buffer (63 mM Tris-HCl (pH = 6.8), 15% glycerol, 2% SDS, 0.15% bromophenol blue, 7 mM DTT) and loaded on a 10% gel followed by SDS-PAGE under reducing conditions. After blotting to a PVDF membrane (Millipore Immobilon Transfer Membrane), the membrane was blocked in 5% milk in TBST (20 mM Tris (pH = 7.4), 150 mM NaCl, 0.05% Tween-20), and was probed with anti-GFP B-2 antibody (Santa Cruz Biotech), washed with TBST, treated with the secondary antibody (goat antimouse horseradish peroxidase-conjugated [Pierce]), and detected using chemiluminescence (SuperSignal West Dura [ThermoScientific]). For ConA (Sigma-Aldrich) detection, the SDS gel was blotted to PVDF membrane and labeled according to manufacturer’s recommendations. To assess protein amounts, the reducing SDS gel was stained with Coomassie Brilliant Blue R250 (Fluka) solution (0.1% Coomassie, 10% glacial acid, 40% methanol) and destained with destaining solution (20% methanol, 10% acetic acid). EndoH (New England Biolabs) digestion was performed according to the manufacturer’s instructions under reducing conditions.

### Confocal Microscopy

Confocal microscopy was previously described [20], with the exception that a Leica SP5 confocal microscope was used.

### Kifunensine Treatment

Whole inflorescences were cut off the plant and incubated in 50 μM Kif solution (Sigma-Aldrich). After 2 d under constant light at 22°C, fluorescence in mature pollen grains was analyzed using a Leica SP5 confocal microscope.

### Eeyarestatin I Treatment

Anthers of flower buds (around stage 11) were dissected and pollen grains were placed on pollen germination media [14] containing various concentrations of EerI (Sigma). The pollen was incubated for 20 h at 22°C in a moisture incubation box. Fluorescence in pollen grains was analyzed using a Leica SP5 confocal microscope.

## Supporting Information

S1 Fig Additional alleles confirm female and male gametophytic phenotypes. (A–D) Aniline Blue staining of callose in PT cell walls 2 DAP. (A) Normal PT reception in a wild-type FG. (B–D) PT overgrowth in *tun-2* (B), *evn-2* (C), and *evn-3* mutant FGs (D). Asterisks mark PT overgrowth phenotype. (E–H) In vitro pollen germination analysis. (E) Normal pollen germination of the wild type. (F) PT bursting phenotype in *tun-2/TUN;qrt/qrt*. Arrows indicate bursting PTs. (G) Degenerated pollen phenotype in *evn-2/EVN;qrt/qrt*. (H) Degenerated pollen phenotype in *evn-3/EVN;qrt/qrt*. Arrowheads indicate degenerated pollen. Scale bars: 20 μm.
(PDF)

S2 Fig Synergid morphology is similar in wild-type, *tun-2*, and *evn-3* embryo sacs. (A–B) FM4-64 staining of membranes in ovules (pistils 2 DAE). (A) Ovule with normal synergid cells. (B) Wild-type ovule with obliquely oriented synergid cells. Abbreviations: CC: central cell, EC: egg cell, Sy: synergid cell, FA: filiform apparatus. (C) Quantification of the two morphological types in wild-type, *tun-2/TUN*, and *evn-3/EVN* ovules.
(PDF)

S3 Fig Callose deposition in *tun* and *evn* ovules is not due to upregulated plant defense responses. RT-PCR of *PLANT DEFENSIN1*.*2* (*PDF1*.*2*), involved in the jasmonate-dependent plant defense response, *PATHOGENESIS RELATED PROTEIN1* (*PR1*), involved in the systemic acquired resistance, *PR5* and *PHENYLALANINE AMMONIA-LYASE1* (*PAL1*), involved in the salicylic acid response, in *evn-1/EVN*, *tun-1/TUN*, *evn-2/EVN*, and wild-type pistils 2 DAE, and in a seedling control. Numbers on the right indicate number of amplification cycles. *ACTIN11* serves as expression control.
(PDF)

S4 Fig Alexander and DAPI staining reveal different pollen phenotypes in *tun* and *evn* mutant pollen grains. (A–C) Alexander staining of mature *qrt/qrt* (A), *tun-1/TUN;qrt/qrt* (B), and *evn-1/EVN;qrt/qrt* pollen tetrads (C). (D–F) DAPI staining of DNA in mature *qrt/qrt* control (D), *tun-1/TUN;qrt/qrt* (E), and *evn-1/EVN;qrt/qrt* pollen tetrads (F). (G) DAPI staining of DNA in stage four (bicellular and early tricellular pollen), stage three (tricellular pollen), and stage two (late tricellular and early mature pollen) *evn-1/EVN;qrt/qrt* mutant tetrads. Scale bars: 20 μm.
(PDF)

S5 Fig Reciprocal crosses reveal that the PT overgrowth phenotype is due to a female gametophytic defect. (A–D) Aniline Blue staining of callose in PT and ovule cell walls 2 DAP. (A) Col-0 ovule pollinated with *tun-1/TUN* pollen. (B) *tun*-1 mutant ovule pollinated with Col-0 pollen. (C) Col-0 ovule pollinated with *evn-1/EVN* pollen. (D) *evn*-1 mutant ovule pollinated with Col-0 pollen. Asterisks indicate PT overgrowth phenotype. Scale bars: 20 μm.
(PDF)

S6 Fig The causative EMS mutation in *evn-1* localizes to the lower arm of chromosome III and disrupts gene *At3g45040*. Ratios of heterozygous SNPs plotted against their chromosomal position. The red dashed line indicates the ratio at 0.5, where the causative SNP is expected. The green dashed line marks the ratio at 0.25, where the unlinked SNPs should locate. The red box indicates the linked and selected region on the lower arm of chromosome III around *At3g45040*. Grey boxes mark centromeric regions with poor mapping quality. Arrow indicates the causative SNP with a segregation ratio of 0.5 in a pool of mutant individuals. The segregation ratio of the *evn-1* allele was as expected due to the high sequence coverage of 130 reads.
(PDF)

S7 Fig The causative EMS mutation in *evn-2* localizes to the lower arm of chromosome III and disrupts gene *At3g45040*. Ratios of heterozygous SNPs plotted against their chromosomal position. The red dashed line indicates the ratio at 0.5, where the causative SNP is expected. The green dashed line marks the ratio at 0.25, where the unlinked SNPs should locate. The red box indicates the linked and selected region on the lower arm of chromosome III around *At3g45040*. Grey boxes mark centromeric regions with poor mapping quality. Arrow indicates the causative SNP with a segregation ratio of 0.3 in a pool of mutant individuals. The segregation ratio of the *evn-2* alleles was lower than expected because of poor sequence coverage (see S1 Text).
(PDF)

S8 Fig TUN-GFP and EVN-GFP translational fusions locate to the endoplasmic reticulum (ER). (A–E) Confocal microscope analysis of fluorescent fusion proteins. (A–C) *pTUN::TUN-GFP* expression in the female gametophyte (A) and the synergids (B) 2 DAE, and in a PT (C). (D) *p35S::TUN-GFP* (left panel) and *p35S::ER-rk* (middle panel) in transiently transformed onion epidermis cell, merged channels (right panel). (E) *p35S::EVN-GFP* (left panel) and *p35S::ER-rk* (middle panel) in transiently transformed tobacco epidermis cells, merged channels (right panel).
(PDF)

S9 Fig *TUN(RNAi)* and *EVN(RNAi)* lines show reduced gene expression. (A) qRT-PCR expression analysis of *TUN* in four independent *RNAi* lines. The corresponding number of dwarfed individuals per line (16 plants) is indicated below. (B) qRT-PCR expression analysis of *EVN* in four independent *RNAi* lines.
(PDF)

S10 Fig FER-GFP in *RNAi(TUN)* lines is not completely deglycosylated. Western blot analysis of FER-GFP protein from control and *TUN(RNAi)* seedlings using an antibody against GFP. Coomassie-stained SDS-PAGE (bottom) serves as control for loaded protein amounts. Asterisk marks completely N-deglycosylated FER-GFP after treatment of the protein extract with the deglycosylase EndoH.
(PDF)

S11 Fig FER-GFP, NTA-GFP, and LRE-Citrine reporters do not differ between wild-type, *tun-2/TUN*, and *evn-3/EVN* pistils. (A) Quantification of the localization of a homozygous FER-GFP reporter in wild-type, *tun-2/TUN*, and *evn-3/EVN* ovules (pistils 2 DAE). Note: Only fluorescent ovules were counted. (B) Quantification of the localization of a hemizygous NTA-GFP reporter in wild-type, *tun-2/TUN*, and *evn-3/EVN* ovules (pistils 2 DAE). Note: Not all ovules display reporter expression. (C) Quantification of the localization of a homozygous LRE-Citrine reporter in wild-type and *evn-3/EVN* ovules (pistils 2 DAE).
(PDF)

S12 Fig Eeyarestatin I treatment of *tun-2/TUN* pollen results in partial ANX1-YFP fluorescence recovery. Relative ANX1-YFP protein abundance in *tun-2/TUN* pollen after treatment with different concentrations of the ERAD inhibitor EerI. Counted pollen grains: 5 μM: *n* = 76; 10 μM: *n* = 222; 15 μM: *n* = 160; 20 μM: *n* = 265; 30 μM: *n* = 147; 50 μM: *n* = 256.
(PDF)

S13 Fig Surveyor nuclease digest of sequenced samples of *evn-1* and *evn-2*. The SNP region of the *At3g45040* gene was amplified from each of the 94 and 60 DNA samples that had been pooled for sequencing from *evn-1* and *evn-2*, respectively, and two Col-0 controls. PCR products were digested with the SURVEYOR nuclease, cleaving single base pair mismatches in heteroduplex DNA [64]. In *evn-1*, the undigested wild-type band is 1,000 bp, whereas any sample containing the SNP displays an undigested band at 1,000 bp and two digestion products at around 800 bp and 200 bp. Individuals six and seven have a wild-type band only, and were shown to be sampling mistakes. In *evn-2*, the undigested wild-type band is 900 bp, whereas any sample containing the SNP displays an undigested band at 900 bp and two digestion products at around 500 bp and 400 bp. Results for *tun-1* were published previously [28].
(PDF)

S1 Table SNP data of the resequenced, EMS-mutagenized genome of *evn-1/EVN*. All data presented has been obtained using the DiBayes algorithm for SNP calling with the highest stringency settings [28].
(XLS)

S2 Table SNP data of the resequenced, EMS-mutagenized genome of *evn-2/EVN*. All data presented has been obtained using the DiBayes algorithm for SNP calling with the highest stringency settings [28].
(XLS)

S3 Table Primers to amplify *evn2* linked genes carrying a non-causative EMS SNP for SURVEYOR analysis. SNP containing regions of 14 genes were amplified. The segregation ratio of the SNP by SRM is indicated in brackets.
(PDF)

S1 Data Quantitative observations for S2C Fig, S9 Fig, S11 Fig and S12 Fig
(XLS)

S1 Text Supporting protocols. (PDF)

## Abbreviations:

ConA: Concanavalin A
CRPs: cysteine-rich polypeptides
*Cr*RLK1L: *Catharanthus roseus* receptor-like kinase 1-like subfamily
DAE: days after emasculation
DAP: days after pollination
DAPI: 4',6-diamidino-2-phenylindole
DEFL: defensin-like protein
Dol-P: phospho-dolichol
EerI: Eeyarestatin I
EFR: *EF*-Tu receptor
EndoH: endoglycosidase H
EMS: ethane methyl sulfonate
ER: endoplasmic reticulum
ERAD: ER-associated degradation
*EVN*: *EVAN*
FA: filiform apparatus
*FER*: *FERONIA*
FG: female gametophyte
*FLS2*: *FLAGELLIN-SENSITIVE2*
GFP: green fluorescent protein
Glc: glucose
GlcNAc: N-acetylglucosamine
GPI: glycosylphosphatidylinositol
GT: glycosyltransferase
GUS: β-glucuronidase
Kif: Kifunensine
*LRE*: *LORELEI*
LRR: leucine-rich repeat
Man: mannose
*MLO*: *MILDEW-RESISTANCE LOCUS O*
*NTA*: *NORTIA*
PT: pollen tube
RLK: receptor-like kinase
RNAi: RNA interference
ROS: reactive oxygen species
SNP: single nucleotide polymorphism
SRM: SNP-ratio mapping
*TUN*: *TURAN*
UDP: uridine diphosphate
WT: wild type
YFP: yellow fluorescent protein.
