# Supplementary figures and images for: TURAN and EVAN Mediate Pollen Tube Reception in Arabidopsis Synergids through Protein Glycosylation
Source: PLoS Biol. 2015 Apr 28;13(4):e1002139. doi: 10.1371/journal.pbio.1002139 (PMC4412406; doi:10.1371/journal.pbio.1002139)

wild type

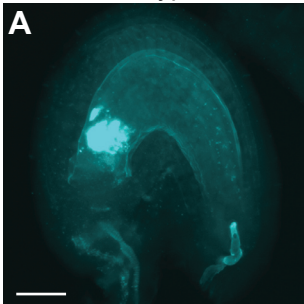*tun-2/TUN*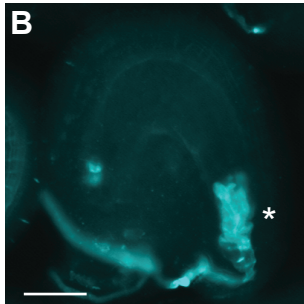*evn-2/EVN*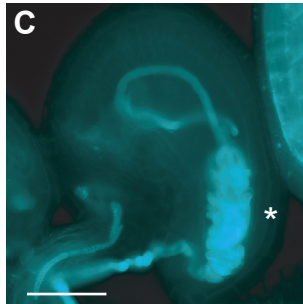*evn-3/EVN*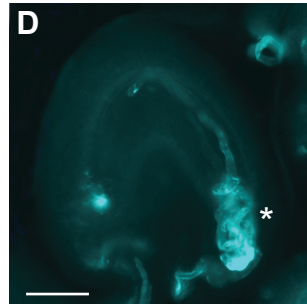

wild type

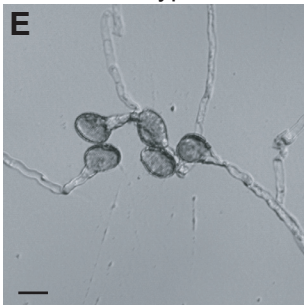*tun-2/TUN;qrt/qrt*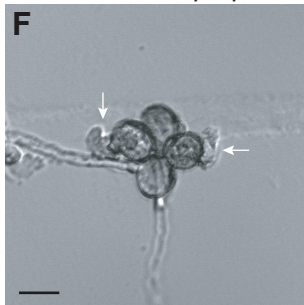*evn-2/EVN;qrt/qrt*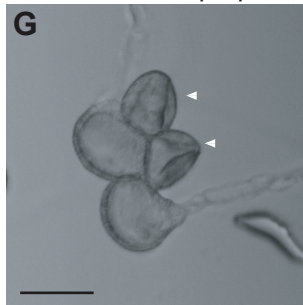*evn-3/EVN;qrt/qrt*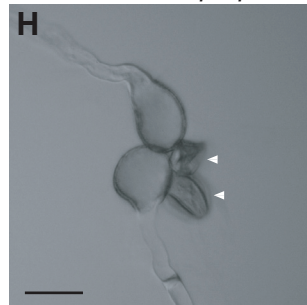

Supplement: S1 Fig — (A–D) Aniline Blue staining of callose in PT cell walls 2 DAP. (A) Normal PT reception in a wild-type FG. (B–D) PT overgrowth in tun-2 (B), evn-2 (C), and evn-3 mutant FGs (D). Asterisks mark PT overgrowth phenotype. (E–H) In vitro pollen germination analysis. (E) Normal pollen germination of the wild type. (F) PT bursting phenotype in tun-2/TUN;qrt/qrt. Arrows indicate bursting PTs. (G) Degenerated pollen phenotype in evn-2/EVN;qrt/qrt. (H) Degenerated pollen phenotype in evn-3/EVN;qrt/qrt. Arrowheads indicate degenerated pollen. Scale bars: 20 μm. (PDF) [file pbio.1002139.s001.pdf]

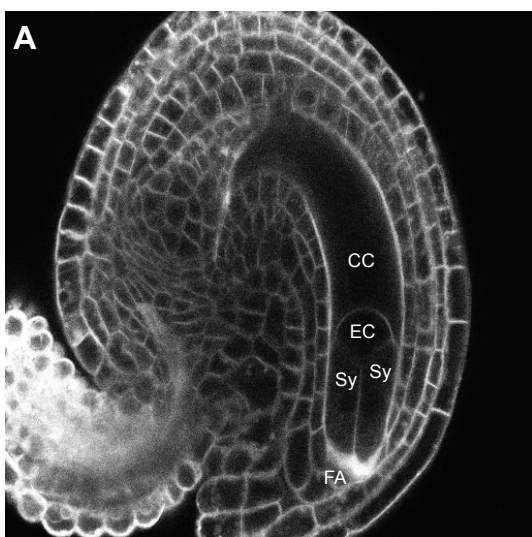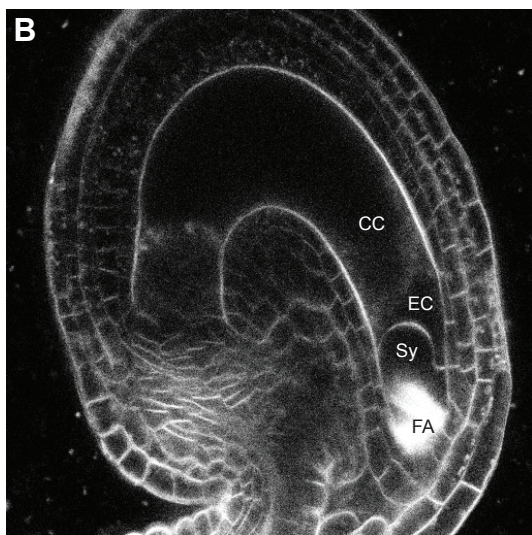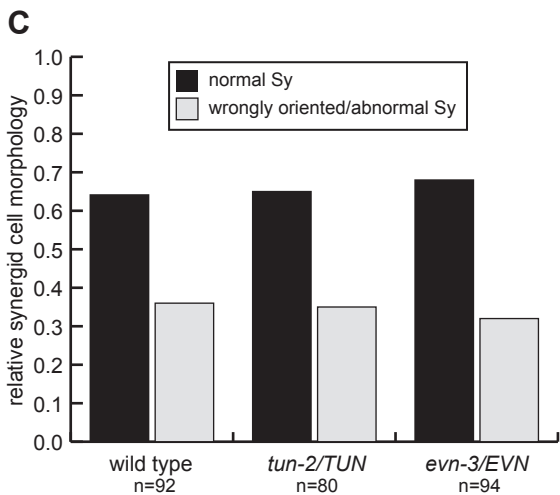

Supplement: S2 Fig — (A–B) FM4-64 staining of membranes in ovules (pistils 2 DAE). (A) Ovule with normal synergid cells. (B) Wild-type ovule with obliquely oriented synergid cells. Abbreviations: CC: central cell, EC: egg cell, Sy: synergid cell, FA: filiform apparatus. (C) Quantification of the two morphological types in wild-type, tun-2/TUN, and evn-3/EVN ovules. (PDF) [file pbio.1002139.s002.pdf]

evn-2  
tun-1  
evn-1  
wild-type pistils  
wild-type seedling  
gDNA

*PDF 1.2*

40x

*PR5*

40x

*PR1*

40x

*PAL1*

40x

*Actin11*

30x

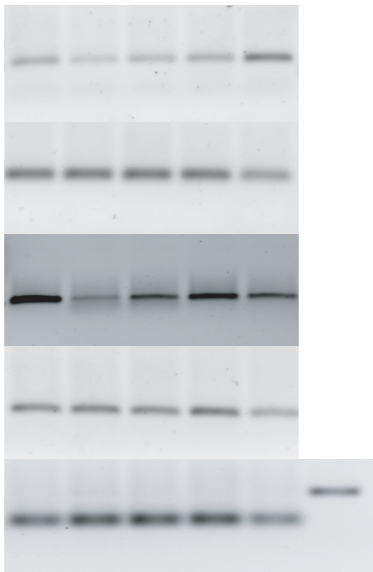

Supplement: S3 Fig — RT-PCR of PLANT DEFENSIN1.2 (PDF1.2), involved in the jasmonate-dependent plant defense response, PATHOGENESIS RELATED PROTEIN1 (PR1), involved in the systemic acquired resistance, PR5 and PHENYLALANINE AMMONIA-LYASE1 (PAL1), involved in the salicylic acid response, in evn-1/EVN, tun-1/TUN, evn-2/EVN, and wild-type pistils 2 DAE, and in a seedling control. Numbers on the right indicate number of amplification cycles. ACTIN11 serves as expression control. (PDF) [file pbio.1002139.s003.pdf]

*evn-1/EVN;qrt/qrt*

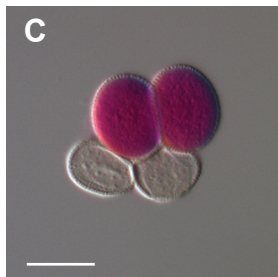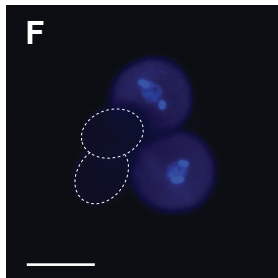

**G** DAPI staining of different pollen stages in *evn-1/EVN;qrt/qrt*

| stage 4 | stage 3 | stage 2 |
|---------|---------|---------|
|         |         |         |

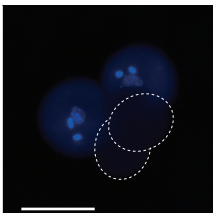

Supplement: S4 Fig — (A–C) Alexander staining of mature qrt/qrt (A), tun-1/TUN;qrt/qrt (B), and evn-1/EVN;qrt/qrt pollen tetrads (C). (D–F) DAPI staining of DNA in mature qrt/qrt control (D), tun-1/TUN;qrt/qrt (E), and evn-1/EVN;qrt/qrt pollen tetrads (F). (G) DAPI staining of DNA in stage four (bicellular and early tricellular pollen), stage three (tricellular pollen), and stage two (late tricellular and early mature pollen) evn-1/EVN;qrt/qrt mutant tetrads. Scale bars: 20 μm. (PDF) [file pbio.1002139.s004.pdf]

Col-0 x *tun-1/TUN*

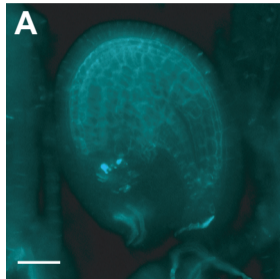

*tun-1/TUN* x Col-0

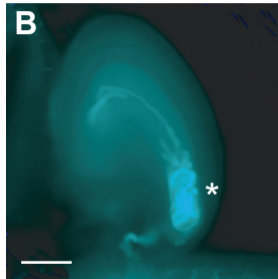

Col-0 x *evn-1/EVN*

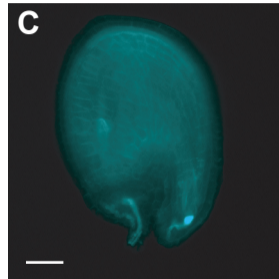

*evn-1/EVN* x Col-0

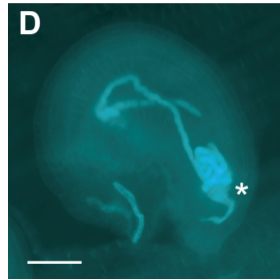

Supplement: S5 Fig — (A–D) Aniline Blue staining of callose in PT and ovule cell walls 2 DAP. (A) Col-0 ovule pollinated with tun-1/TUN pollen. (B) tun-1 mutant ovule pollinated with Col-0 pollen. (C) Col-0 ovule pollinated with evn-1/EVN pollen. (D) evn-1 mutant ovule pollinated with Col-0 pollen. Asterisks indicate PT overgrowth phenotype. Scale bars: 20 μm. (PDF) [file pbio.1002139.s005.pdf]

Chromosome I

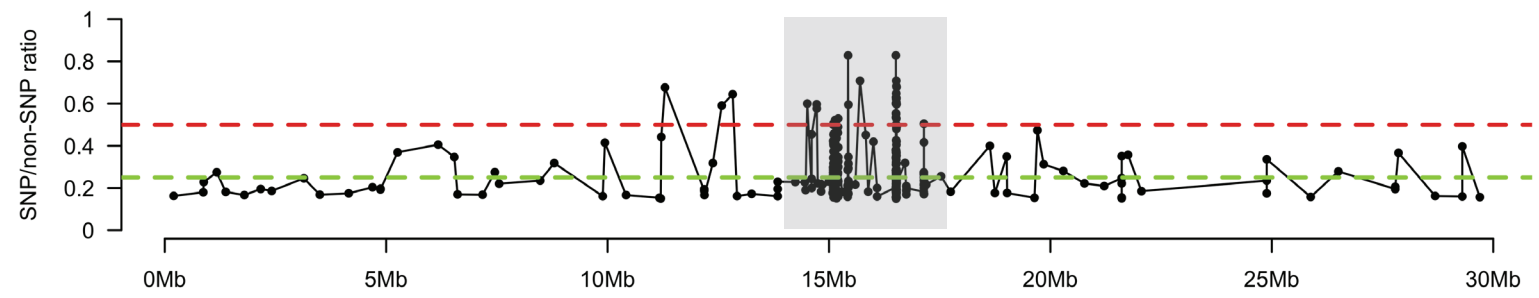

Chromosome II

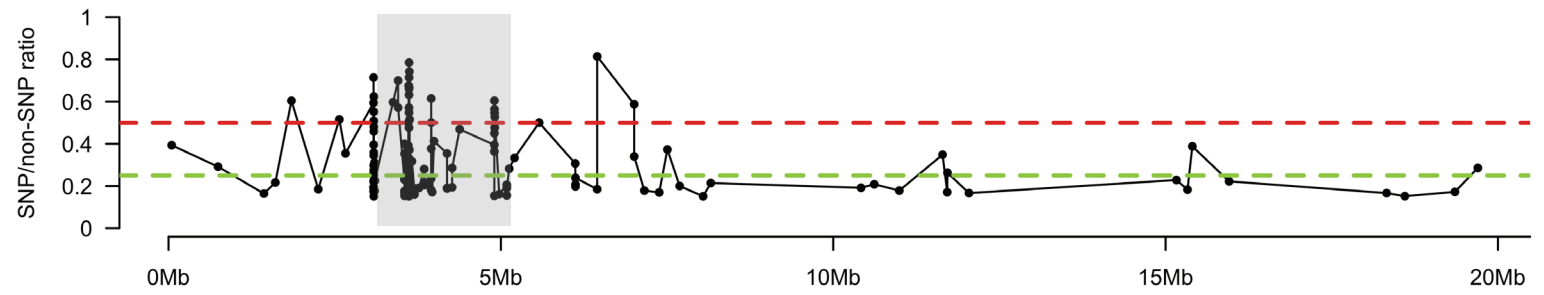

Chromosome III

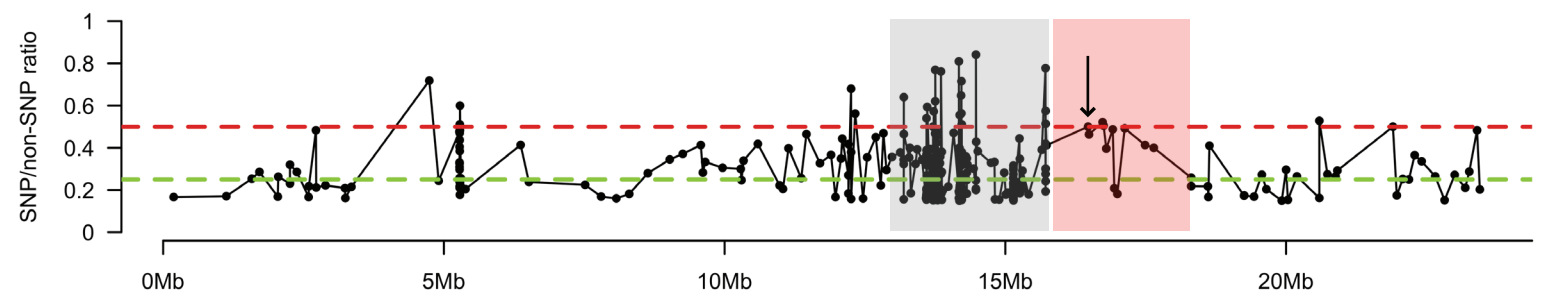

Chromosome IV

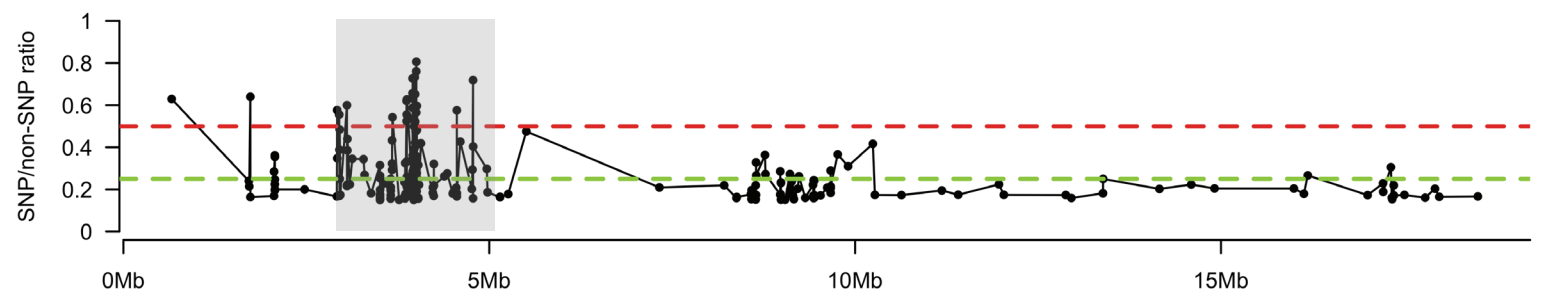

Chromosome V

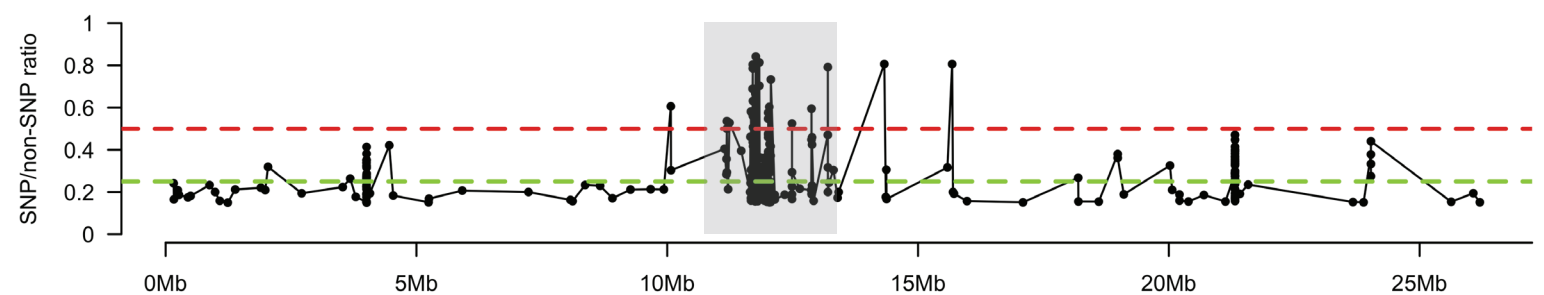

Supplement: S6 Fig — Ratios of heterozygous SNPs plotted against their chromosomal position. The red dashed line indicates the ratio at 0.5, where the causative SNP is expected. The green dashed line marks the ratio at 0.25, where the unlinked SNPs should locate. The red box indicates the linked and selected region on the lower arm of chromosome III around At3g45040. Grey boxes mark centromeric regions with poor mapping quality. Arrow indicates the causative SNP with a segregation ratio of 0.5 in a pool of mutant individuals. The segregation ratio of the evn-1 allele was as expected due to the high sequence coverage of 130 reads. (PDF) [file pbio.1002139.s006.pdf]

Chromosome I

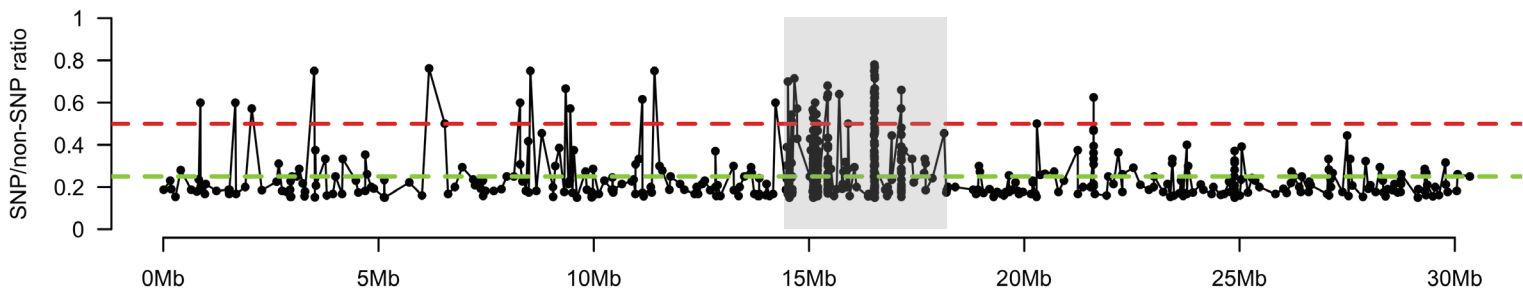

Chromosome II

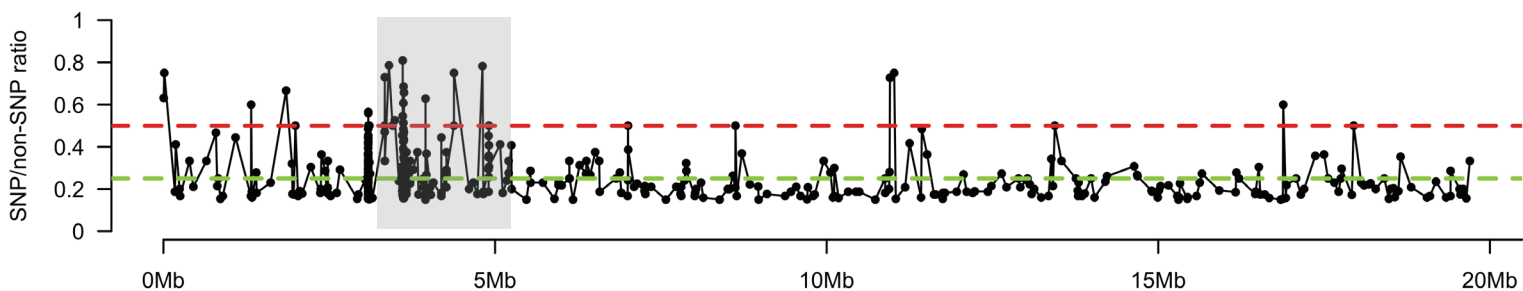

Chromosome III

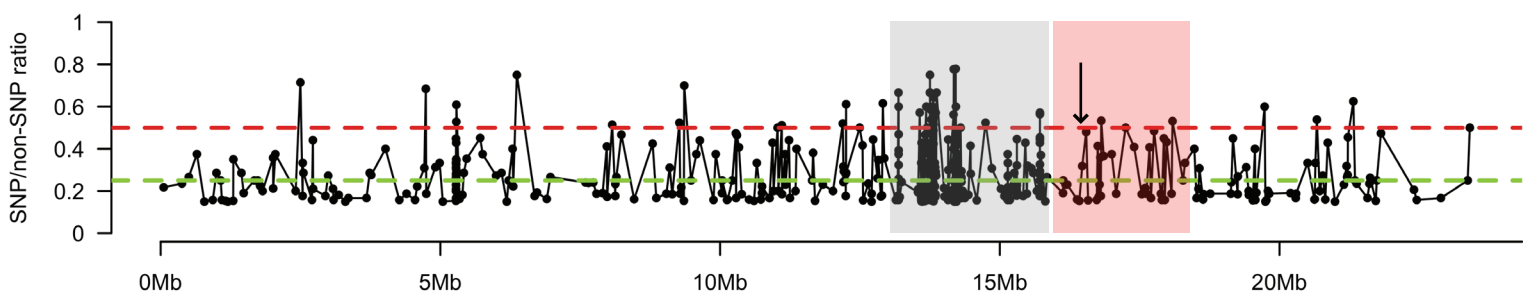

Chromosome IV

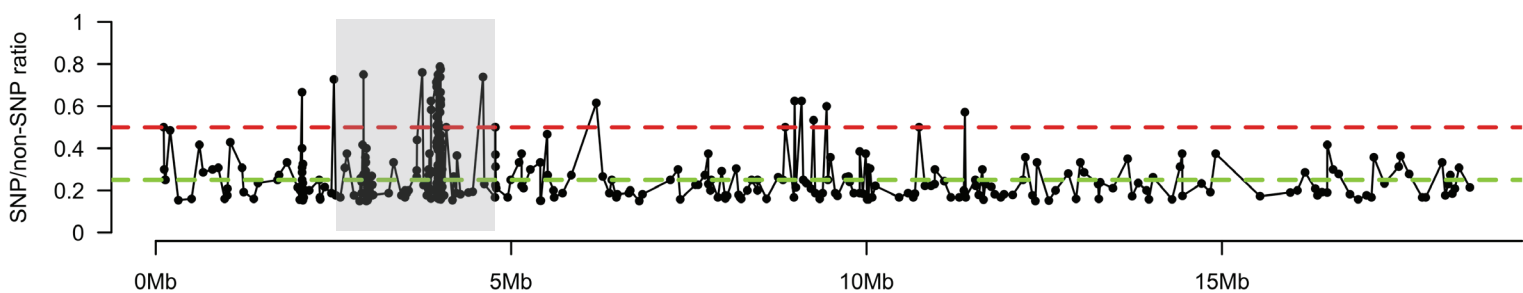

Chromosome V

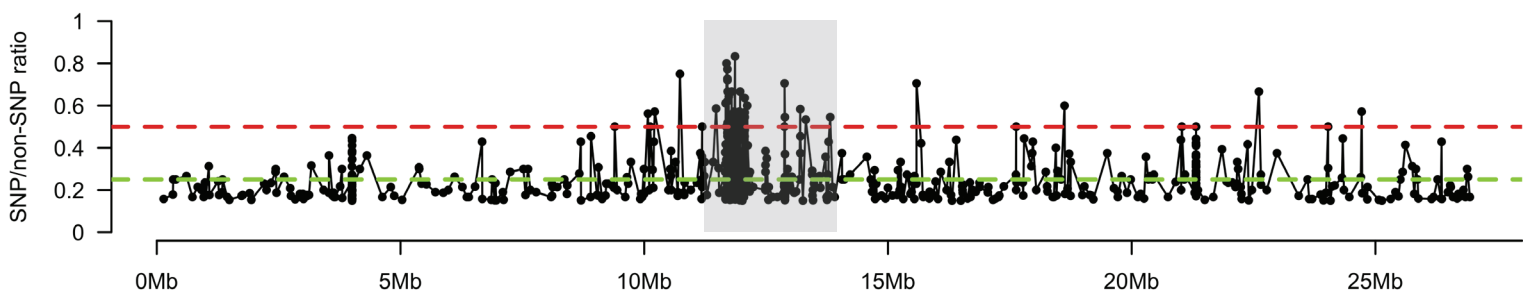

Supplement: S7 Fig — Ratios of heterozygous SNPs plotted against their chromosomal position. The red dashed line indicates the ratio at 0.5, where the causative SNP is expected. The green dashed line marks the ratio at 0.25, where the unlinked SNPs should locate. The red box indicates the linked and selected region on the lower arm of chromosome III around At3g45040. Grey boxes mark centromeric regions with poor mapping quality. Arrow indicates the causative SNP with a segregation ratio of 0.3 in a pool of mutant individuals. The segregation ratio of the evn-2 alleles was lower than expected because of poor sequence coverage (see S1 Text). (PDF) [file pbio.1002139.s007.pdf]

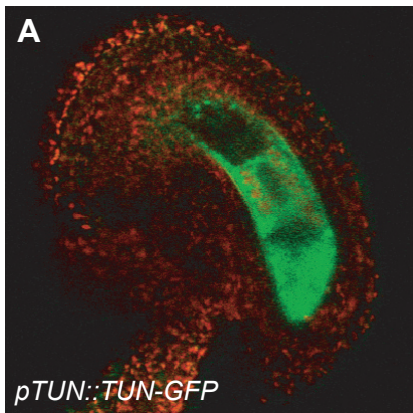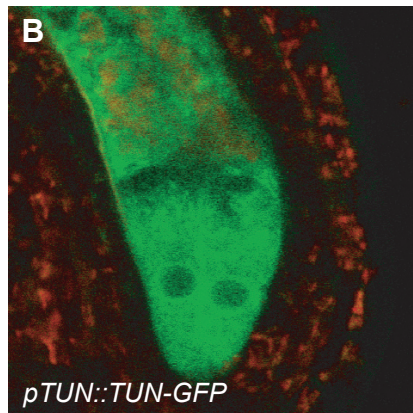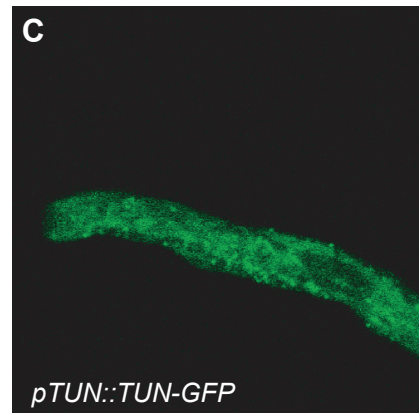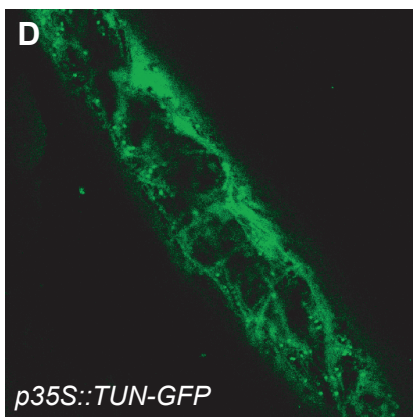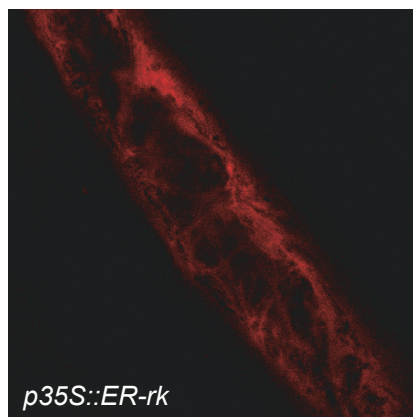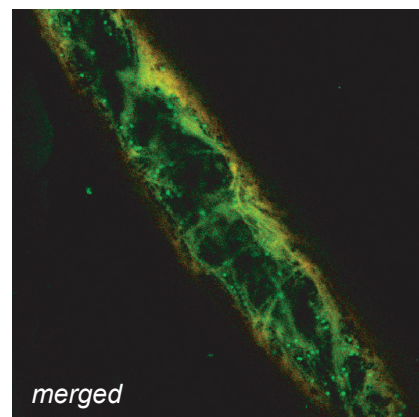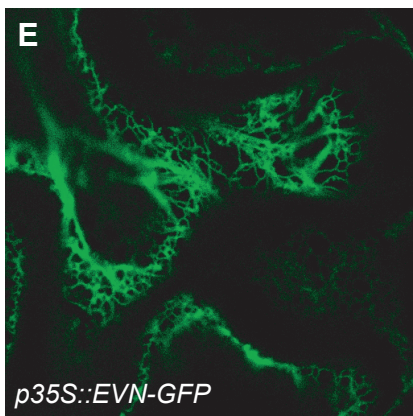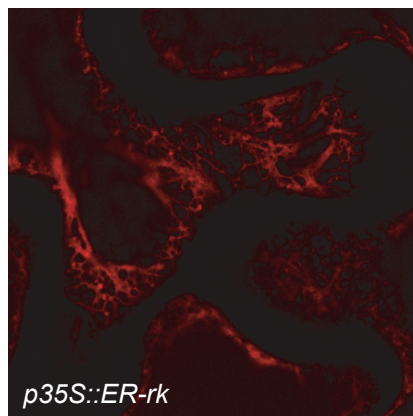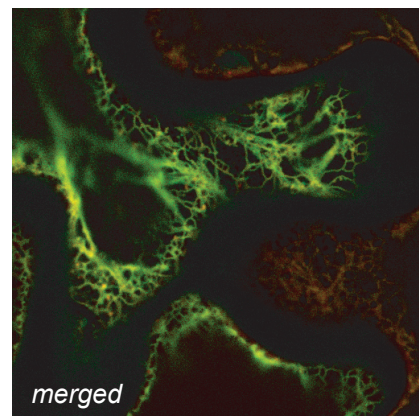

Supplement: S8 Fig — (A–E) Confocal microscope analysis of fluorescent fusion proteins. (A–C) pTUN::TUN-GFP expression in the female gametophyte (A) and the synergids (B) 2 DAE, and in a PT (C). (D) p35S::TUN-GFP (left panel) and p35S::ER-rk (middle panel) in transiently transformed onion epidermis cell, merged channels (right panel). (E) p35S::EVN-GFP (left panel) and p35S::ER-rk (middle panel) in transiently transformed tobacco epidermis cells, merged channels (right panel). (PDF) [file pbio.1002139.s008.pdf]

A

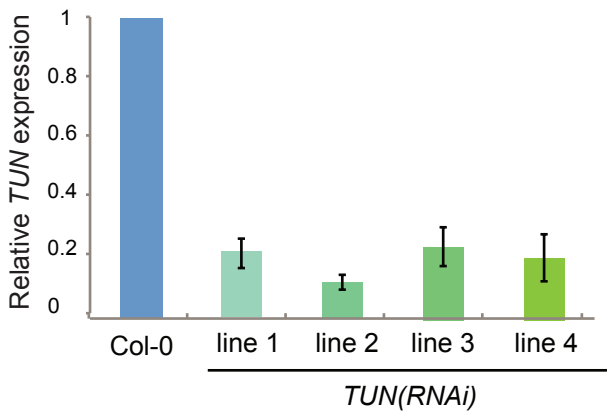

dwarfs / 16 plants

0

11

13

4

11

B

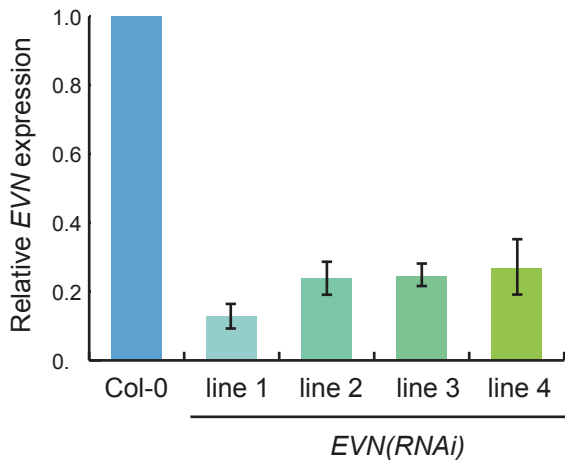

Supplement: S9 Fig — (A) qRT-PCR expression analysis of TUN in four independent RNAi lines. The corresponding number of dwarfed individuals per line (16 plants) is indicated below. (B) qRT-PCR expression analysis of EVN in four independent RNAi lines. (PDF) [file pbio.1002139.s009.pdf]

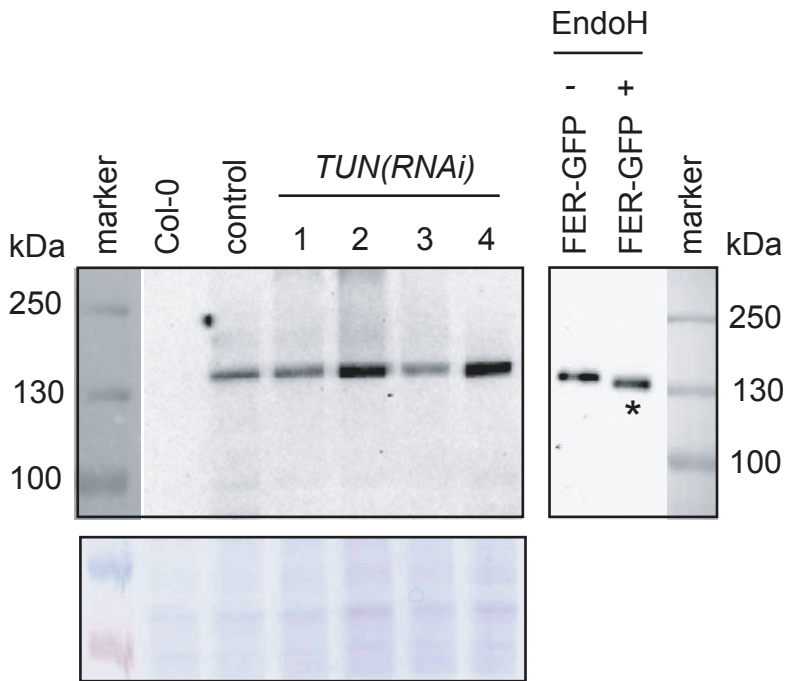

Supplement: S10 Fig — Western blot analysis of FER-GFP protein from control and TUN(RNAi) seedlings using an antibody against GFP. Coomassie-stained SDS-PAGE (bottom) serves as control for loaded protein amounts. Asterisk marks completely N-deglycosylated FER-GFP after treatment of the protein extract with the deglycosylase EndoH. (PDF) [file pbio.1002139.s010.pdf]

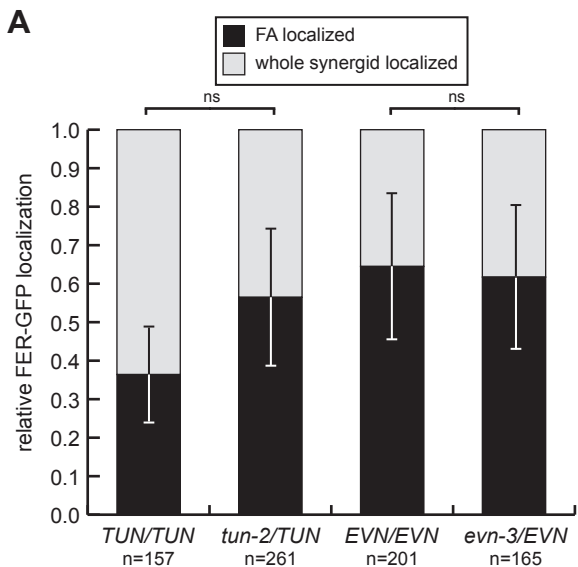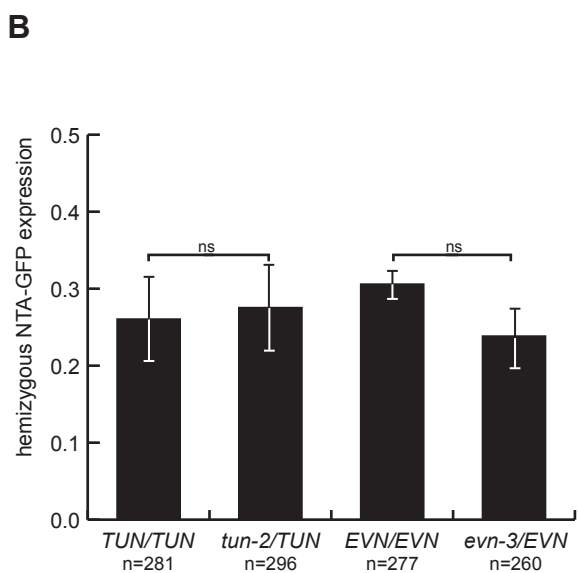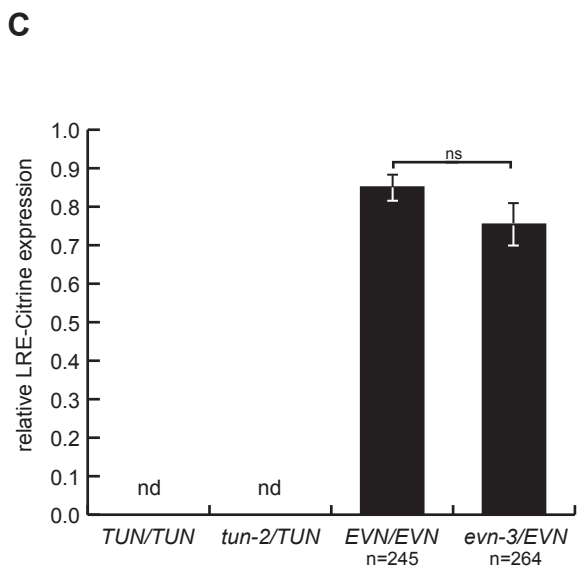

Supplement: S11 Fig — (A) Quantification of the localization of a homozygous FER-GFP reporter in wild-type, tun-2/TUN, and evn-3/EVN ovules (pistils 2 DAE). Note: Only fluorescent ovules were counted. (B) Quantification of the localization of a hemizygous NTA-GFP reporter in wild-type, tun-2/TUN, and evn-3/EVN ovules (pistils 2 DAE). Note: Not all ovules display reporter expression. (C) Quantification of the localization of a homozygous LRE-Citrine reporter in wild-type and evn-3/EVN ovules (pistils 2 DAE). (PDF) [file pbio.1002139.s011.pdf]

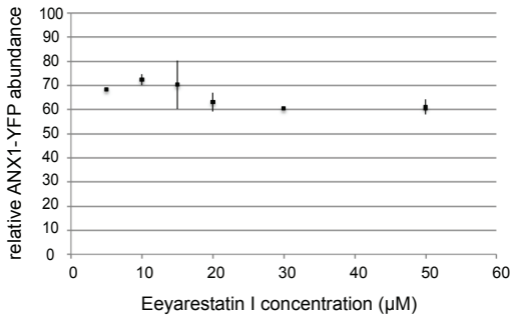

Supplement: S12 Fig — Relative ANX1-YFP protein abundance in tun-2/TUN pollen after treatment with different concentrations of the ERAD inhibitor EerI. Counted pollen grains: 5 μM: n = 76; 10 μM: n = 222; 15 μM: n = 160; 20 μM: n = 265; 30 μM: n = 147; 50 μM: n = 256. (PDF) [file pbio.1002139.s012.pdf]
