# Supplementary material for: TURAN and EVAN Mediate Pollen Tube Reception in Arabidopsis Synergids through Protein Glycosylation
Source: PLoS Biol. 2015 Apr 28;13(4):e1002139. doi: 10.1371/journal.pbio.1002139 (PMC4412406; doi:10.1371/journal.pbio.1002139)

A Surveyor assay for *AT3G45040* in the *evn-1* SRM mutant sequencing population

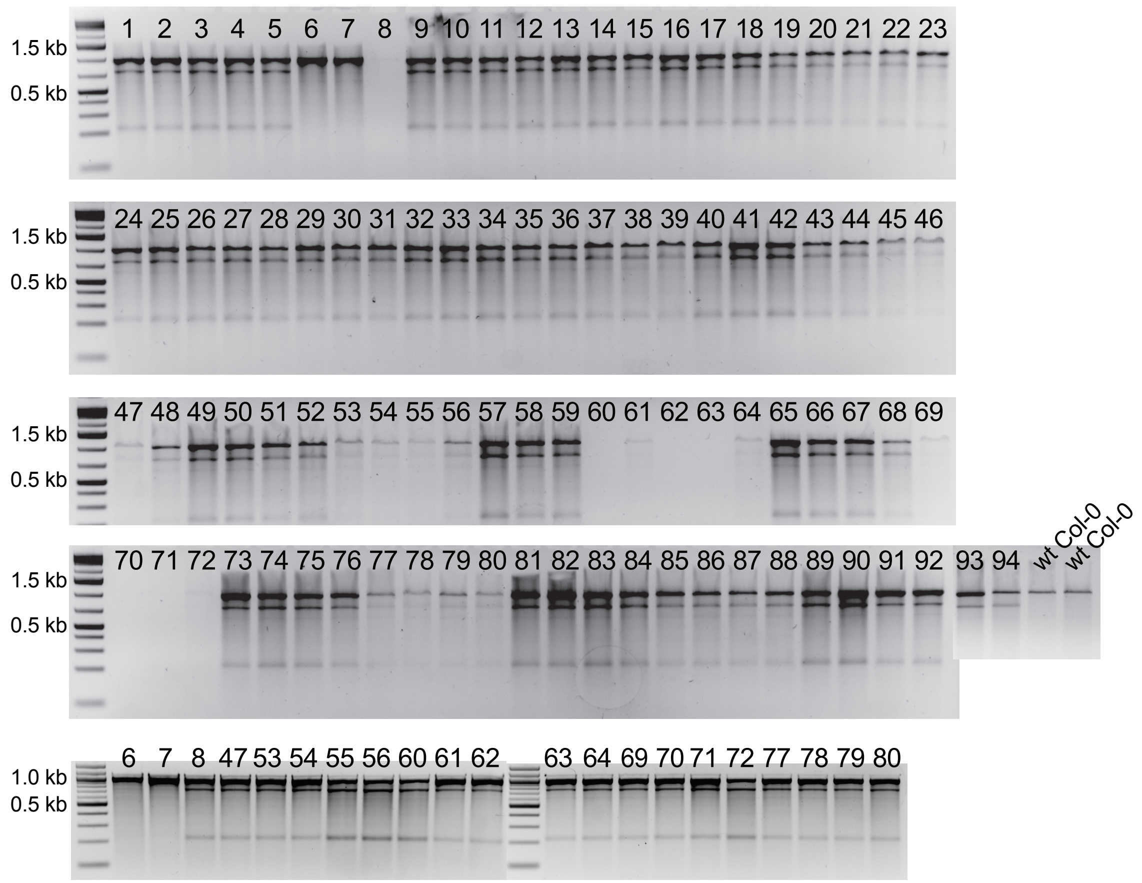

B Surveyor assay for *AT3G45040* in the *evn-2* SRM mutant sequencing population

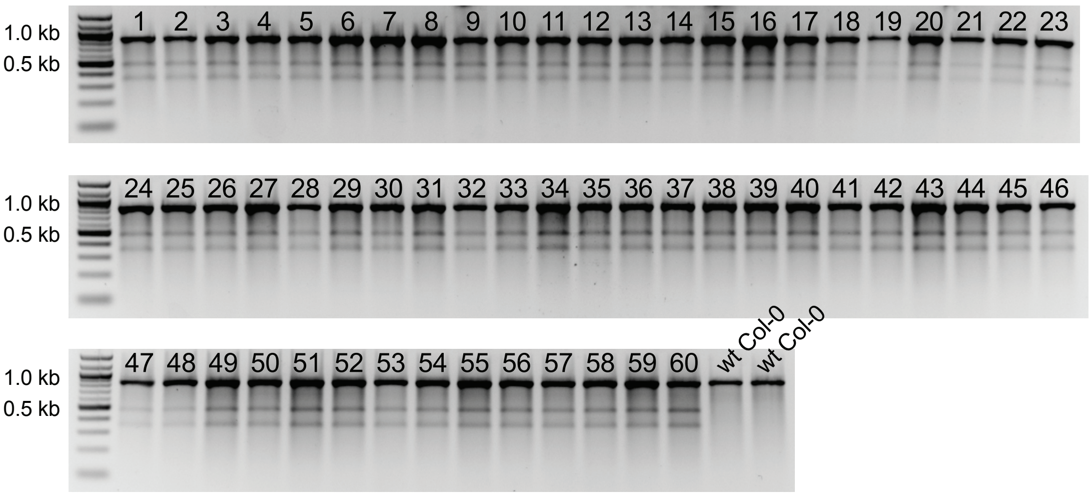

Supplement: S13 Fig — The SNP region of the At3g45040 gene was amplified from each of the 94 and 60 DNA samples that had been pooled for sequencing from evn-1 and evn-2, respectively, and two Col-0 controls. PCR products were digested with the SURVEYOR nuclease, cleaving single base pair mismatches in heteroduplex DNA [64]. In evn-1, the undigested wild-type band is 1,000 bp, whereas any sample containing the SNP displays an undigested band at 1,000 bp and two digestion products at around 800 bp and 200 bp. Individuals six and seven have a wild-type band only, and were shown to be sampling mistakes. In evn-2, the undigested wild-type band is 900 bp, whereas any sample containing the SNP displays an undigested band at 900 bp and two digestion products at around 500 bp and 400 bp. Results for tun-1 were published previously [28]. (PDF) [file pbio.1002139.s013.pdf]
