# Supplementary material for: TURAN and EVAN Mediate Pollen Tube Reception in Arabidopsis Synergids through Protein Glycosylation
Source: PLoS Biol. 2015 Apr 28;13(4):e1002139. doi: 10.1371/journal.pbio.1002139 (PMC4412406; doi:10.1371/journal.pbio.1002139)
Supplement: S3 Table — SNP containing regions of 14 genes were amplified. The segregation ratio of the SNP by SRM is indicated in brackets. (PDF) [file pbio.1002139.s016.pdf]

At3G56230 (ratio 0.43):
Forward 5'-CTTGGAGCGTTATGGCGTAT-3'
Reverse 5'-TGCACCACAATGCCATCTAT-3'
At3G59020 (ratio 0.47):
Forward 5'-GCCATAAGCTGCACATGAGA-3'
Reverse 5'-GGTTTGTGCCAAAACTTGGT-3'
AT3G58410 (ratio 0.24):
Forward 5'-CTGATCAAACCAGCGATGTG-3'
Reverse 5'-AAGTCACGAGGGACAAATGC-3'
At3G51570 (ratio 0.19):
Forward 5'-GCTCACATTGTCCTCAGCAA-3'
Reverse 5'-GATTTGGTGGAAGCTTTGGA-3'
At3G52320 (ratio 0.31) :
Forward 5'-TGCGTGACCAGTTCTCAAAG-3'
Reverse 5'-CTTGTGAGAGCCGCAACATA-3'
At3G55690 (ratio 0.54):
Forward 5'-GGGCAGGAATTACGGATTTT-3'
Reverse 5'-TATTTCCGGGGAAGGAAAAC-3'
At3G52660 (ratio 0.16):
Forward 5'-CCTCCACATGGTTCAGAGGT-3'
Reverse 5'-AGCCGAAGAATCTCCTCCTC-3'
At3G55640 (ratio 0.33):
Forward 5'-ACGAGGCTTCACGGATATTG-3'
Reverse 5'-CAAGAAAGCAAAAGGCCAAC-3'
At3G57290 (ratio 0.32):
Forward 5'-GGTTTAACGGCGATGAAAAA-3'
Reverse 5'-CAAGAGCGTCAGCTTTACCC-3'
At3G57310 (ratio 0.27):
Forward 5'-TGATGCAGATTTCATTGTTGG-3'
Reverse 5'-TCTTTGAATTTCTGCAAGTCTGA-3'
At3G43180 (ratio 0.16):
Forward 5'-AAAGCGATGTGGGAACAAAG-3'
Reverse 5'-GCTCAAGCTGTTCCTCTTGC-3'
At3G45630 (ratio 0.41):
Forward 5'-AACCATTTTGACTGCCAAGC-3'
Reverse 5'-GTTGCATGGATGGGGATAAC-3'
At3G45850 (ratio 0.37):
Forward 5'-TAGCTTTCTTCCCGCTCTCA-3'
Reverse 5'-TTCGAAACATTCGCATGAAC-3'
At3G48090 (ratio 0.5):
Forward 5'-ATCGCTGGCTTGAGAAGTGT-3'
Reverse 5'-ACAAGGAAGAAGCAGGAGCA-3'
