# Supplementary material for: TURAN and EVAN Mediate Pollen Tube Reception in Arabidopsis Synergids through Protein Glycosylation
Source: PLoS Biol. 2015 Apr 28;13(4):e1002139. doi: 10.1371/journal.pbio.1002139 (PMC4412406; doi:10.1371/journal.pbio.1002139)
Supplement: S1 Text — (PDF) [file pbio.1002139.s018.pdf]

SUPPORTING PROTOCOLS

Aniline Blue Staining of Ovule Sections

For callose staining in semi-thin sections, flower buds were emasculated and harvested 2 DAE. Pistils were fixed in 3:1 Ethanol:Acetic acid under vacuum for 2x10min, and incubated for 12 hours at 4°C. The fixative was replaced by 70% Ethanol and subsequently embedded into Paraplast X-tra embedding medium (Sigma-Aldrich), using the embedding machine Leica ASP200 as previously described [1]. Sectioning was as described before [1], with the exception that we made 6 µm sections. Samples were dewaxed and dehydrated after incubation for 1h at room temperature in 2x 10min Histoclear (100%), 2 x 2min EtOH (100%), followed by 2min of 80%, 60%, 30% EtOH each, 2min 0.75% NaCl and 2min H_2O treatment. Aniline Blue staining was performed using 0.1% Methyl Blue (Sigma-Aldrich), and samples were analyzed with a Leica SP5 confocal microscope.

Pollen Analysis

Anthers from different stages were collected and analyzed by Alexander staining as described [2]. For DAPI staining, anthers were fixed in 3:1 Ethanol:Acetic acid over night at 4°C and stored in 70% EtOH. For analysis 1µg/ml DAPI (4',6-diamidino-2-phenylindole) solution (0.1M sodium phosphate (pH 7), 1 mM EDTA, 0.1% Triton X-100, 1µg/ml DAPI; high grade, Sigma-Aldrich) was added and the sample was analyzed using a Leica DM6000B epifluorescence microscope.

Identification of *tun-1*, *evn-1* and *evn-2*

tun-1, *evn-1* and *evn-2* EMS-alleles were identified by SNP-ratio mapping (SRM; [3]). SNP data of *evn-1* and *evn-2* resequencing are presented in S1 Table and S2 Table, respectively. Sequencing coverage for *evn-2* was only 30x and thus too low to identify the causative SNP by SRM (S7 Fig.). Therefore, rough mapping of an F2 mapping population was done after outcrossing *evn-2/EVN* to the Landsberg *erecta* accession. Linkage was found for marker

ciw4 (70cM) on the lower arm of chromosome 3, and several candidate genes from SRM in this region were tested by the Surveyor nuclease digest (see below). Primers used for gene amplification of non-causative SNPs are found in S3 Table. To confirm the causative mutations in *evn-1* and *evn-2*, the SNP containing region was amplified from every individual in the sequencing pool using primers:

evn-1: 5'-TGGGCCCAACCAATATAAAG-3' and 5'-TCTCAGCCTAGGAATTGGAGA-3';

evn-2: 5'- GCCAATATGGGCCTTGTTAT-3' and 5'-TGACCATCGTGACTCAGAGC-3'.

The PCR products were digested with Surveyor Cel-A nuclease (Transgenomics), according to manufacturer`s recommendations, in order to determine whether the SNP was present in all mutants. Surveyor assays are shown in S13 Fig.

Constructs for Stable Transformation

All PCR reactions for cloning were done using Phusion HF polymerase (NEB), Phusion HF PCR buffer, and a final concentration of 2mM $MgCl_2$, 0.2mM dNTPs and 0.2-0.4mM primer. If not mentioned otherwise, we used the following standard PCR program: 28 to 34 cycles (94ºC for 15 sec, 58ºC for 20 sec, and 72ºC for 30-120 sec), followed by 72°C for 5min. Gateway cloning was performed as described in [4] and only gene-specific primers containing the first 12 base pairs of the attB sites are mentioned below.

TUN-GFP and EVN-GFP fusion protein constructs: For Gateway cloning of *pTUN::TUN-GFP*, the complete coding sequence, without the stop-codon but including a 462bp long promoter region of *TUN* (*At1g16570*), was amplified using gene-specific primers including attB Gateway sites: 5'-AAAAAGCAGGCTTAGCGTCTCACAATCTCC-3' and 5'-AGAAAGCTGGGTGTGAATCTGCAATTTGAGA-3'. For Gateway cloning of *pEVN::EVN-GFP*, the complete coding sequence without the stop codon, including a 1272bp long promoter region of *EVN* (*At3g45040*), was amplified using gene-specific primers including attB Gateway sites: 5'- AAAAAGCAGGCTCACAACAGAAGAATTATC-3' and 5'-AGAAAGCTGGGTGCAAGCAGAGGAGTGAGTA-3'. For Gateway cloning of *p35S::TUN-GFP* and *p35EVN::EVN-GFP*, the complete coding sequence, without the stop codon, was

amplified by gene-specific primers including attB Gateway sites: *TUN:* 5'-AAAAAGCAGGCTTAATGGGGAAAAGAGGAAGGGC-3' and 5'-AGAAAGCTGGGTGTGAATCTGCAATTTGAGA-3'; *EVN:* 5'-AAAAAGCAGGCTTAATGAAGACGACGGCGACG-3' and 5'-AGAAAGCTGGGTGCAAGCAGAGGAGTGAGTA-3'. PCR products were cloned via BP reaction into the pDONR207, and subsequently via LR reaction into the destination vectors (pMDC107 for native-promoter constructs and pMDC83 for 35S-promoter constructs [5]) according to the manufacturer's recommendation (Invitrogen). *pTUN::TUN-GFP* and *pEVN::EVN-GFP* were transformed (see below) into *tun-2/TUN* and *evn-2/EVN* plant lines, respectively. Progeny were selected on MS plates containing 25mg/L Hygromycin to select for transformants.

LRE-Citrine fusion protein constructs: Cloning of the *pLRE::LRE-Citrine* construct was done using the Gibson cloning Master Mix from New England Biolabs (NEB) according to the manufacturer`s recommendations. This method is based on overlapping PCR products. Thus, a 779bp long promoter sequence with the predicted signal peptide from *LRE* [6] was amplified with primers containing an overhang for the vector pMDC99 [5]: 5'-GTGCTGCAAGGCGATTAAGTCCGTGTGCTCTGTCTGCATT-3' and 5'-CACAGCTCCACCTCCACCTCCAGGCCGGCCTATGGAACTTGAAGAGGAGAGAGA-3'.*Citrine* was amplified from the transgenic line CS36962 from the *Arabidopsis* Biological Recourse Center (ABRC), using gene-specific primers with an overhang for the signal peptide of *LRE* and an overhang for the GPI-anchor of *LRE*: 5`-GGCCGGCCTGGAGGTGGAGGTGGAGCTGTGAGCAAGGGCGAGGAGCT-3' and 5'-GGCCCCAGCGGCCGCAGCAGCACCAGCAGGATCCTTGTACAGCTCGTCCA-3'. The GPI-anchor of *LRE* was amplified with overhang primers for the pMDC99 vector: 5'-TGCTGGTGCTGCTGCGGCCGCTGGGGCCTCGGGTATGTCTTTTTGTTGTC-3' and 5'-AGCTCCACCGCGGTGGCGGCCGCTCTAGAAGTCTCGCTTCTTCTTTTGT-3'. pMDC99 was amplified with overhangs for the *LRE* promoter and the GPI-anchor, using the primers: 5'-ACTTAATCGCCTTGCAGCAC-3' and 5'- TCTAGAGCGGCCGCCACCGCGG-3'.

pLRE::LRE-Citrine was transformed to *tun-2/TUN* and *evn-2/EVN* lines. Progeny were selected on MS plates containing 25mg/L Hygromycin to select for transformants.

FER-GFP, ANX1-YFP, ANX2-YFP fusion protein constructs*:* The *pFER::FER-GFP, pACA9::ANX1-YFP*, and *pACA9::ANX2-YFP* constructs were described previously [7,8] and were transformed into *Arabidopsis* Col-0 wild-type and *tun-2/TUN* mutant plants, respectively. Progeny were selected on MS plates containing 25mg/L Hygromycin to select for transformants.

TUN(RNAi) and *EVN(RNAi)* constructs*:* For *TUN* downregulation by RNAi, exon 13 was amplified using primers with attB overhangs for Gateway cloning: 5'-AAAAAGCAGGCTCATTCAAGAACTCGTTAAAG -3' and 5'-AGAAAGCTGGGTCTGAGTGATTAAAGGTTTTGC-3'. For *EVN* downregulation, exon 13 including the 3`UTR was amplified using primers with attB overhangs for Gateway cloning: 5'- AAAAAGCAGGCTAGAAAACGGTTGAAGGAACAGC-3' and 5'-AGAAAGCTGGGTATACGGCAATTAACAAGAGG-3'. PCR products were cloned via BP reaction in pDONR207 and subsequently via LR reaction into destination vector pHellsgate12 [9], according to the manufacturer's recommendation (Invitrogen). *pFER::FER-GFP* expressing Col-0 plants (described above) were transformed using the transformation technique described below. Progeny were selected on MS plates containing 50mg/L Kanamycin and 25mg/L Hygromycin to select transformants for the silencing construct and *FER-GFP*, respectively.

Transformation Techniques

E. coli transformations were conducted using competent *E. coli* strain DH5-alpha F'I^q from NEB. For plant transformation, the destination vector was transformed into competent *Agrobacterium tumefaciens* strain GV3101 [10], and *Arabidopsis thaliana* transformation was done by floral dip [11].

Transient Expression in Onion and Tobacco Epidermis Cells

Transient onion epidermis transformation was previously described [12]. For tobacco leaf infiltration, a 5ml overnight culture of *Agrobaterium tumefaciens* strain GV3101 [10], containing the plasmid of interest, was spun down for 15min 4000rpm (Eppendorf Centrifuge 5804R with the A-4-44 rotor) and dissolved in 2ml AS-medium (3M $MgCl_2$, 1M MES-Buffer, pH=5.6, 150mM Acetosyringone). The OD was adjusted to 0.8 using a photometer (BioRad SmartSpec3000), and different clones were mixed equally, and incubated 2-4h shaking at 28°C. 3 week old *Nicotiana benthamiana* plants were watered before infiltration, and leaves were infiltrated abaxially using a standard syringe without a needle. Infiltrated plants were incubated for 2-3 days and small leave pieces were analyzed using a Leica SP5 confocal microscope. The control ER-marker pER-rk (mCherry) was obtained from ABRC [13].

DNA Extraction and Genotyping

DNA was extracted as previously described [14]. T-DNA insertion lines were genotyped using three primers in one PCR reaction as follows: The wild-type allele of *TUN* using primers 5'-TCCGACTCATTAGCGTCTCAC-3' and 5'-CTGGACCAAGAAAATGTCAGG-3', and the wild-type allele of *EVN* using primers: 5'-TTCGCAGTCATGAAATGGTC-3' and 5'-TGAGAATTCCAGCAAATGGA-3'. The T-DNA insertion was amplified using the T-DNA-specific primer (LB1-Syg) 5'-GCCTTTTCAGAAATGGATAAATAGCCTTGC-3'.

After identification of the causative EMS SNPs, genotyping of *tun-1/TUN*, *evn-1/EVN* and *evn-2/EVN* was done using dCAPS primers. For *tun-1/TUN* the primers 5'-GGTTATTATCTATGAGAAATTTCATCTCACCTCACAGC-3' and 5'-GGAAAGATGGCAACAGGTTC-3' were used to amplify the gene; a subsequent digest with *Alu*I (NEB) revealed mutant individuals by cutting the mutant PCR product. *evn-1/EVN* was amplified using the primers 5'-TCTCCAATTCCTAGGCTGAGAATGC-3' and 5'-TTCTCACTCTTGCTTGGGTGT-3' to amplify the gene; a subsequent digest with *Nhe*I (NEB) revealed mutant individuals by cutting the mutant PCR product. For *evn-2/EVN* genotyping

the primers 5’-CGGTGGCTGTCACAGCCACCAGAAGCGAGCG-3’ and 5’-GATGGCAGTGTGCTTTGTTC-3’ were used to amplify the gene; a subsequent digest with *Hha*I (NEB) revealed mutant individuals by cutting the mutant PCR product.

RNA Extraction, RT-PCR and qRT-PCR

To investigate upregulation of defense-related genes in mutant siliques and residual expression of *TUN* and *EVN* in the RNAi knockdown lines, RNA from 25 emasculated *tun-1/TUN*, *evn-1/EVN*, *evn-2/EVN* and Col-0 pistils, and 2-3 cauline leaves of RNAi plants and Col-0 control plants, respectively, was extracted using the Trizol reagent (Invitrogen) according to the manufacturer`s recommendations. cDNA was reverse transcribed using Oligo-dT primers and Superscript II reverse transcriptase (Invitrogen) according to the manufacturer`s recommendations.

RT-PCR analysis of defense-related genes was done using the following primers: *PR1*: 5’-TCGGAGCTACGCAGAACAACT-3’ and 5’-TCGGAGCTACGCAGAACAACT-3’; *PDF1.2*: 5'-TGGTGGAAGCACAGAAGTTG-3' and 5'-GATCCATGTTTGGCTCCTTC-3'; *PR5*: 5’-TCCTTGACCGGCGAGAGTT-3’ and 5’- AGGAACAATTGCCCTACCACC-3’; *PAL1*: 5’-AACGGAGGAGGAGTGGACG-3’ and 5’- CTTTCATTTGCTCCGCTGC-3’; *Actin11*: 5'-AACTTTCAACACTCCTGCCATG-3' and 5'-CTGCAAGGTCCAAACGCAGA-3'. Primers for *PR1*, *PDF1.2*, *PR5* and *PAL1* were previously published [15,16].

qRT-PCR was performed using a real-time PCR system (Applied Biosystems 7500 Fast Real-Time PCR System) with the SYBR green qPCR mix (Applied Biosystems PCR Master Mix) according to manufacturer’s recommendation. qRT-PCR of *TUN* was done using the primers 5’-TCAGAAGAGCAACACCATTATCCC-3’ and 5’-CTGCGAGCCACATTGTACGG-3’. qRT-PCR of *EVN* was done using the primers 5’-GACCGAGCCTTATCTCCATTTGC-3’ and 5’- ATGTTATTCCCGCTGCTGTTCC-3’. Expression levels were normalized against *UBIQUITIN C* (*UBC*, primers: 5’-ATGCTTGGAGTCCTGCTTGG-3’ and 5’-TGCCATTGAATTGAACCCTCTC-3’).

SUPPORTING REFERENCES

1. Wuest SE, Vijverberg K, Schmidt A, Weiss M, Gheyselinck J, et al. (2010) *Arabidopsis* female gametophyte gene expression map reveals similarities between plant and animal gametes. Curr Biol 20: 506–512.

2. Alexander MP (1969) Differential staining of aborted and nonaborted pollen. Biotech Histochem 44: 117-122.

3. Lindner H, Raissig MT, Sailer C, Shimosato-Asano H, Bruggmann R, et al. (2012) SNP-Ratio Mapping (SRM): Identifying lethal alleles and mutations in complex genetic backgrounds by next-generation sequencing. Genetics 191: 1381–1386.

4. Raissig MT, Bemer M, Baroux C, Grossniklaus U (2013) Genomic imprinting in the *Arabidopsis* embryo is partly regulated by PRC2. PLoS Genet 9: e1003862.

5. Curtis MD, Grossniklaus U (2003) A gateway cloning vector set for high-throughput functional analysis of genes *in planta*. Plant Phys 133: 462–469.

6. Capron A, Gourgues M, Neiva LS, Faure JE, Berger F, et al. (2008) Maternal control of male-gamete delivery in *Arabidopsis* involves a putative GPI-anchored protein encoded by the *LORELEI* gene. Plant Cell 20: 3038–3049.

7. Escobar-Restrepo JM, Huck N, Kessler S, Gagliardini V, Gheyselinck J, et al. (2007) The *FERONIA* receptor-like kinase mediates male-female interactions during pollen tube reception. Science 317: 656–660.

8. Boisson-Dernier A, Lituiev DS, Nestorova A, Franck CM, Thirugnanarajah S, et al. (2013) ANXUR receptor-like kinases coordinate cell wall integrity with growth at the pollen tube tip via NADPH oxidases. PLoS Biol 11: e1001719. doi:10.1371/journal.pbio.1001719.s023.

9. Helliwell CA, Waterhouse PM (2005) Constructs and methods for hairpin RNA-mediated gene silencing in plants. Meth Enzymol 392: 24–35.

10. Koncz C, Schell J (1986) The promoter of TL-DNA gene 5 controls the tissue-specific expression of chimaeric genes carried by a novel type of *Agrobacterium* binary vector. Mol Gen Genet 204: 383-396.

11. Zhang X, Henriques R, Lin S-S, Niu Q-W, Chua N-H (2006) *Agrobacterium*-mediated transformation of *Arabidopsis thaliana* using the floral dip method. Nat Protoc 1: 641–646.

12. Varagona MJ, Schmidt RJ, Raikhel NV (1992) Nuclear localization signal(s) required for nuclear targeting of the maize regulatory protein Opaque-2. Plant Cell 4: 1213–1227.

13. Nelson BK, Cai X, Nebenführ A (2007) A multicolored set of *in vivo* organelle markers for co-localization studies in *Arabidopsis* and other plants. Plant J 51: 1126–1136.

14. Edwards K, Johnstone C, Thompson C (1991) A simple and rapid method for the preparation of plant genomic DNA for PCR analysis. Nucleic Acids Res 19: 1349.

15. Beckers GJM, Jaskiewicz M, Liu Y, Underwood WR, He SY, et al. (2009) Mitogen-

activated protein kinases 3 and 6 are required for full priming of stress responses in *Arabidopsis thaliana*. Plant Cell 21: 944–953.

16. Qiu JL, Zhou L, Yun BW, Nielsen HB, Fiil BK (2008) *Arabidopsis* mitogen-activated protein kinase kinases *MKK1* and *MKK2* have overlapping functions in defense signaling mediated by *MEKK1*, *MPK4*, and *MKS1*. Plant Phys 148: 212-222.
